# Survey of the green picoalga *Bathycoccus* genomes in the global ocean

**DOI:** 10.1038/srep37900

**Published:** 2016-11-30

**Authors:** Thomas Vannier, Jade Leconte, Yoann Seeleuthner, Samuel Mondy, Eric Pelletier, Jean-Marc Aury, Colomban de Vargas, Michael Sieracki, Daniele Iudicone, Daniel Vaulot, Patrick Wincker, Olivier Jaillon

**Affiliations:** 1 CEA - Institut de Génomique, GENOSCOPE, 2 rue Gaston Crémieux, 91057 Evry, France; 2 CNRS, UMR 8030, CP5706 Evry, France; 3 Université d’Evry, UMR 8030, CP5706 Evry, France; 4 Sorbonne Universités, UPMC Université Paris 06, CNRS, UMR7144, Station Biologique de Roscoff, 29680 Roscoff, France; 5 National Science Foundation, 4201 Wilson Blvd., Arlington, VA 22230, USA; 6 Stazione Zoologica Anton Dohrn, Villa Comunale, 80121 Naples, Italy

## Abstract

*Bathycoccus* is a cosmopolitan green micro-alga belonging to the Mamiellophyceae, a class of picophytoplankton that contains important contributors to oceanic primary production. A single species of *Bathycoccus* has been described while the existence of two ecotypes has been proposed based on metagenomic data. A genome is available for one strain corresponding to the described phenotype. We report a second genome assembly obtained by a single cell genomics approach corresponding to the second ecotype. The two *Bathycoccus* genomes are divergent enough to be unambiguously distinguishable in whole DNA metagenomic data although they possess identical sequence of the 18S rRNA gene including in the V9 region. Analysis of 122 global ocean whole DNA metagenome samples from the *Tara*-Oceans expedition reveals that populations of *Bathycoccus* that were previously identified by 18S rRNA V9 metabarcodes are only composed of these two genomes. *Bathycoccus* is relatively abundant and widely distributed in nutrient rich waters. The two genomes rarely co-occur and occupy distinct oceanic niches in particular with respect to depth. Metatranscriptomic data provide evidence for gain or loss of highly expressed genes in some samples, suggesting that the gene repertoire is modulated by environmental conditions.

Phytoplankton, comprising prokaryotes and eukaryotes, contribute to nearly half of the annual global primary production^1^. Picocyanobacteria of the genera *Prochlorococcus* and *Synechococcus* dominate the prokaryotic component^2^. However, small eukaryotes (picoeukaryotes; <2 μm) can be major contributors to primary production^3^,^4^. In contrast to cyanobacteria, the phylogenetic diversity of eukaryotic phytoplankton is wide, with species belonging to virtually all photosynthetic protist groups^5^. Among them, three genera of green algae belonging to the order Mamiellales (class Mamiellophyceae^6^), *Micromonas, Ostreococcus* and *Bathycoccus* are particularly important ecologically because they are found in a wide variety of oceanic ecosystems, from the poles to the tropics^7^,^8^,^9^,^10^,^11^,^12^. The cosmopolitan distribution of these genera raises the questions of their diversity and their adaptation to local environmental conditions. These genera exhibit genetic diversity: for example, there are at least three genetically different clades of *Micromonas* with different habitat preferences^12^,^13^. One ecotype of *Micromonas* seems to be restricted to polar waters^8^,^14^. *Ostreococcus* which is the smallest free-living eukaryotic cell known to date with a cell size of 0.8 μm^15^ can be differentiated into at least four clades. Two *Ostreococcus* species have been formerly described: *O. tauri* and *O. mediterreanus*^15^,^16^. Among these *Ostreococcus* clades, different strains seem to be adapted to different light ranges^17^. However, the ecological preferences of *Ostreococcus* strains are probably more complex, implying other environmental parameters such as nutrients and temperature^9^.

The genus *Bathycoccus* was initially isolated at 100 m from the deep chlorophyll maximum (DCM) in the Mediterranean Sea^18^ and cells with the same morphology (body scales) had been reported previously from the Atlantic Ocean^19^. *Bathycoccus* has been since found to be widespread in the oceanic environment, in particular in coastal waters^20^,^21^, and one genome sequence from a coastal strain is available^22^. Metagenomic data have suggested the existence of two *Bathycoccus* ecotypes^10^,^11^,^23^, recently named B1 and B2^11^. These two ecotypes have identical 18S rRNA sequences and therefore cannot be discriminated when using metabarcodes such as the V4 or V9 regions of the 18S rRNA genes^10^. However information on the ocean-wide distribution and the ecological preferences of these two ecotypes are lacking.

Mapping of metagenomic reads onto whole genomes (fragment recruitment) has been shown to be an efficient way to assess the distribution of oceanic bacterial populations^24^,^25^. The paucity of eukaryotic genomes and metagenomes has prevented this approach to be applied on a large scale to eukaryotes. Therefore the determination of the geographical distribution and ecological preferences of marine eukaryotic species has relied on the use of marker genes such as 18S rRNA or ITS (internal transcribed spacer)^26^ and more recently on metabarcodes^27^. One major problem is the absence of reference genomes for many marine eukaryotes as a consequence of the difficulty to cultivate them. To overcome this limitation, Single Cells Genomics is a very promising approach^28^,^29^. However, this approach has been largely used for bacteria^30^ and numerous technical challenges have limited the recovery of eukaryotic genomes with this approach^28^,^31^,^32^,^33^. The most complete assembly obtained so far is for an uncultured stramenopile belonging to the MAST-4 clade and contains about one third of the core eukaryotic gene set^33^. Recently, the *Tara* Oceans expedition collected water samples from the photic zone of hundreds of marine sites from all oceans and obtained physicochemical parameters, such as silicate, nitrate, phosphate, temperature and chlorophyll^34^,^35^,^36^. This expedition also led to the massive sequencing of the V9 region from 18S ribosomal gene providing a description of the eukaryotic plankton community over wide oceanic regions^27^. During this expedition a large number of metagenomic data and single-cell amplified genomes (SAGs^37^) have also been acquired. Here, we introduce a novel genome assembly for *Bathycoccus* based on the sequence assembly of four SAGs obtained from a *Tara* Oceans sample collected in the Arabian Sea. Comparison of this assembly with the reference sequence of *Bathycoccus* strain RCC1105^22^ unravels substantial genomic divergence. We investigated the geographical distributions of these two genomes by mapping onto them the short reads of a large set of metagenomes obtained in multiple marine basins from the *Tara* Oceans survey^35^,^38^. We also determined the genomic properties and habitat preferences of these two *Bathycoccus*.

## Results

### Genome structure of *Bathycoccus* TOSAG39-1

We obtained a new *Bathycoccus* SAG assembly (TOSAG39-1) by the single cell genomics approach from four single cells collected from a single sample during the *Tara* Oceans expedition. We presumed these cells were from the same population and combined their genomic sequences to improve the assembly. The length of the final combined-SAGs assembly is 10.3 Mb comprising 2 345 scaffolds. Half of the assembled genome lies in 179 scaffolds longer than 13.6 kb (N50 size). This assembly covers an estimated 64% of the whole genome when considering the proportion of identified eukaryotic conserved genes^39^. We verified that this combined SAG assembly has longer cumulative size, and a larger representation of the genome than each assembly obtained from sequences of a single-SAG. We also merged the four assemblies from single-SAGs and, after removing redundancies, we obtained a substantially lower genomic representation than for the combined-SAGs strategy (Table 1). We mapped the reads of each SAG-sequencing onto the final assembly to examine whether genomic variability among the sampled population might have affected the quality of the assembly. We did not detect any major genomic variability; contigs can be formed by reads from different cells (Supplementary Figure S1). In total, half of the assembly (52.2%) was generated by reads from a single cell and one third (30.5%) by two cells.

The approximate estimated genome size is 16 Mb and GC content is 47.2%, similar to what has been reported for RCC1105 (15 Mb and 48%, respectively). We predicted 6 157 genes (Supplementary Table 1), representing a higher gene density compared to RCC1105 (622 *vs.* 520 genes per Mb), probably because of the higher fragmentation of the SAG assembly (the coding base density is conversely higher in TOSAG39-1, 742 *vs.* 821 kb/Mb for the two assemblies, respectively, Supplementary Table 1). The photosynthetic capacity of TOSAG39-1, presumed from the chlorophyll autofluorescence in the cell sorting step, was verified by the presence of plastid contigs (removed during quality control filtering) and by the presence of nuclear photosynthetic gene families (encoding RuBisCo synthase, starch synthase, alternative oxidase and chlorophyll a/b binding proteins) in the final assembly.

Previous comparisons of Mamiellales genomes demonstrated global conservation of chromosomal locations of genes between *Bathycoccus, Ostreococcus* and *Micromonas*^22^. These genera all possess outlier chromosomes (one part of chromosome 14 and the entire chromosome 19 for *Bathycoccus*) that display an atypical GC% and numerous small, unknown, non-conserved genes. We detected almost perfect co-linearity between non-outlier chromosomes of RCC1105 and orthologous regions of TOSAG39-1 scaffolds (Supplementary Figure S2). However, there is a significant evolutionary divergence between the genomes: the orthologous proteins are only 78% identical on average (Supplementary Figure S3). Only 26 genes are highly conserved (>99% identity), they are distributed on 14 chromosomes (including outlier chromosome 14) and did not display any clustering. As expected, chromosome 19 did not fit this pattern: we could not align most of its genes by direct BLAST comparison. Some traces of homology were observed for nine genes (62% protein identity). One of the twenty longest scaffolds of TOSAG39-1 had characteristics similar to chromosome 19. This scaffold could not be aligned to RCC1105 and has the lowest GC content (0.44 *vs.* 0.48% for the other scaffolds on average).

Manual curation of alignments to analyze synteny along the twenty longest TOSAG39-1 scaffolds showed that 90% of genes are collinear between the two genomes, 5% are shared outside syntenic blocks, and 5% are specific to TOSAG39-1. The three rRNA genes (18S or small subunit (SSU), 5S, 23S or large subunit (LSU)), used as phylogenetic markers in many studies, are identical between the two genomes. The SSU and LSU genes of TOSAG39-1 have introns. The SSU intron (440 bp) is at the same position as in RCC1105, but is only 91% similar. The LSU intron (435 bp) is only present in TOSAG39-1. The internal transcribed spacers (ITS) are different between the two TOSAG39-1 and the RCC1105 assemblies (82% and 86% for ITS1 and ITS2, respectively) but closer to those of two *Bathycoccus* oceanic strains from the Indian Ocean (RCC715 and RCC716) (Supplementary Figure S4) and of a metagenome from the Atlantic Ocean DCM^40^. We also looked at the plastid 16 S marker gene^41^ and to the PRP8 intein gene that has been proposed as markers for *Bathycoccus*^10^. The plastid 16 S sequences of the two *Bathycoccus* genomes share 92% identical nucleotides, and PRP8 is lacking from the TOSAG39-1 assembly.

We were able to determine the affiliation of three metagenomes^23^,^40^ containing *Bathycoccus* and two *Bathycoccus* transcriptomes of the MMETSP database^42^ (Supplementary Figures S5). Metagenomes T142 and T149 from the South East Pacific^23^ and transcriptome MMETSP1399 (strain CCMP1898, which is the type strain for *Bathycoccus prasinos*) correspond, or are closely related to RCC1105. The tropical Atlantic Ocean metagenome^40^ and transcriptome MMETSP1460 (strain RCC716 from the Indian Ocean) correspond, or are closely related to TOSAG39-1. Direct amino acid BLAST^43^ comparison of TOSAG39-1 and RCC1105 versus metagenomes T142 and T149 demonstrates the presence of additional genomes in these samples that were obtained by flow cytometry sorting of natural picoplankton populations (Supplementary Figure S5).

### Oceanic distribution of *Bathycoccus* genomes

**Figure 1:**
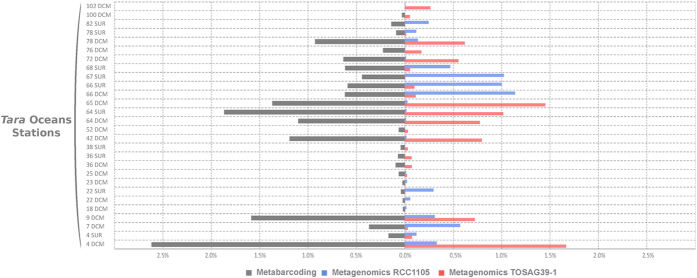
Comparisons of relative abundances of *Bathycoccus* in the 0.8–5 μm size fraction samples from *Tara* Oceans stations. Left: relative 18S rRNA V9 amplicons abundance (percent of reads). Right: relative metagenomic abundances (percent of metagenomic reads) from direct mapping of metagenomic reads onto two genome sequence assemblies (strain RCC1105 and TOSAG39-1, single cell assembly from an Indian Ocean sample). Stations and depth (Surface or DCM) are indicated on the Y axis.

We analyzed the worldwide distribution of the two *Bathycoccus* genomes using metagenomic samples from the *Tara* Oceans expedition. Metagenomic short reads obtained from 122 samples taken at 76 sites and covering 24 oceanic provinces were mapped onto the two *Bathycoccus* genomes RCC1105 and TOSAG39-1. Among the four eukaryotic size fractions sampled in this expedition (0.8–5 μm, 5–20 μm, 20–180 μm, 180–2000 μm) statistically significant mapping was only obtained for the 0.8–5-μm fraction, which matches the cellular size of *Bathycoccus* (1.5–2.5 μm^18^). The percentage of filtered mapped metagenomic reads for every gene and station was used to estimate the relative genomic abundance of *Bathycoccus.* We compared final counts of genome abundances with counts based on amplicon sequences of the V9 region of the 18S rRNA gene^27^ which does not distinguish RCC1105 from TOSAG39–1 because their 18S rRNA gene sequences are identical. The V9 data demonstrated the wide distribution of *Bathycoccus* in marine waters, with maximum relative abundance reaching 2.6% of all reads. The *Bathycoccus* metabarcode was represented by more than 1% of reads in 13% of the samples. *Bathycoccus* sequences were detected in whole metagenome reads from the same samples where *Bathycoccus* was detected with 18S rRNA metabarcodes (Fig. 1). For each sample displaying a V9 signal, we detected the presence of the genomes of either RCC1105, TOSAG39-1, or both. In addition, the relative abundances estimated from V9 metabarcodes were correlated with the sum of the relative genomic abundances of TOSAG39-1 and RCC1105 (Supplementary Figure S6). Therefore, the *Bathycoccus* populations detected by the V9 metabarcode are likely to correspond to these two genomes only, and not to a third yet unknown genome.

Among the 58 samples where *Bathycoccus* metagenomics abundances represented more than 0.01% of the total numbers of reads, in 91% of the cases a single genome was dominant, i.e. accounting for more than 70% of the reads. The two *Bathycoccus* showed similar proportions (i.e., between 40% and 60% of the reads) in only two samples (stations TARA_006 and TARA_150 at DCM, Supplementary Figure S7).

**Figure 2:**
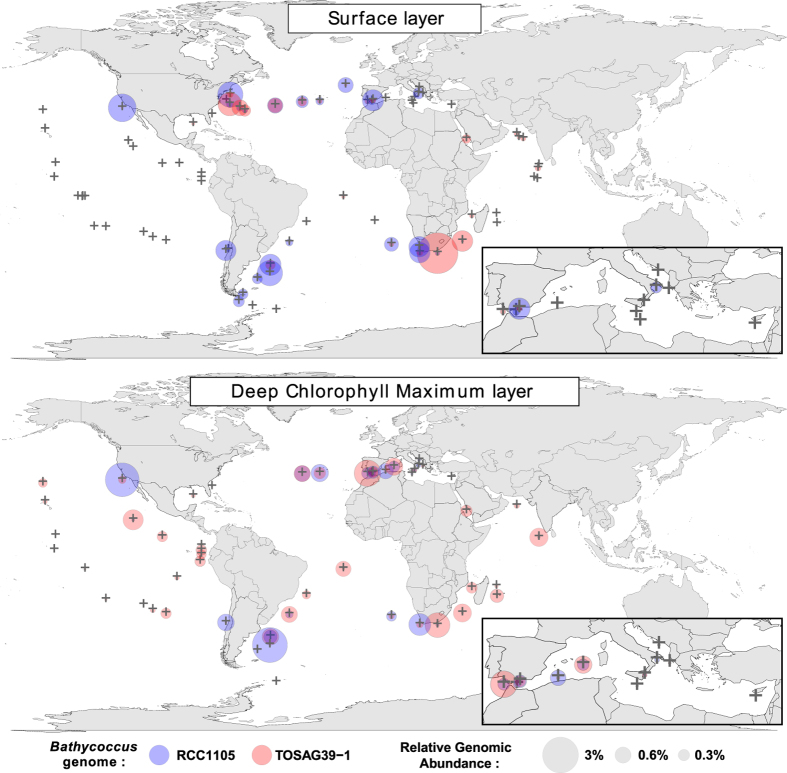
Geographical distribution of two *Bathycoccus* genomes, RCC1105 and TOSAG39-1, along *Tara* Oceans expedition stations from recruitments of metagenomic reads. Top and bottom maps correspond to the surface and deep chlorophyll maximum (DCM) samples respectively. Gray crosses indicate *Tara* Oceans sampling stations and the sizes of the red or blue circles indicate the relative genomic abundances of the two *Bathycoccus* types. We generated this map using R-package maps_2.1-6, mapproj_1.1-8.3, gplots_2.8.0 and mapplots_1.4 (version R-2.13, https://cran.r-project.org/web/packages/maps/index.html).

The global distribution of the two *Bathycoccus* genomes revealed complex patterns. The RCC1105 genome was found mainly in temperate waters, both at the surface and at the DCM, whereas TOSAG39-1 appeared more prevalent in tropical zones and at the DCM (Fig. 2). TOSAG39-1 was found in surface water in only five winter samples from the Agulhas and Gulf Stream regions at stations undergoing strong vertical mixing (Supplementary Table 2, Supplementary Figure S8). RCC1105 was detected more widely in surface water and was restricted to two narrow latitudinal bands around 40°S and 40°N. Conversely, TOSAG39-1 was found throughout a latitudinal range from 40°S to 39°N (Fig. 2). In particular, TOSAG39-1 was found in the tropical and subtropical regions in the Pacific, Atlantic and Indian Oceans.

In the equatorial and tropical Pacific Ocean, a region characterized by high nutrient and low chlorophyll where phytoplankton is limited by iron^44^, *Bathycoccus* was not detected (or only at very low abundance), except close to the Galapagos Islands. We detected opposite trends in the presence of the two *Bathycoccus* along the Gulf Stream: RCC1105 increased from west to east while TOSAG39-1 showed the reverse trend. The two *Bathycoccus* also showed opposite trends at some stations that were relatively close but located on both sides of important oceanographic boundaries. The first case was off South Africa, between stations TARA_065 and TARA_066 (Supplementary Figure S8) located, respectively, in coastal, temperate Atlantic and in Indian subtropical water from the Agulhas current^45^.

The second case occurred in winter in the North Atlantic, downstream of Cape Hatteras (US East coast), where station TARA_145 was in cold, nutrient-rich waters north of the northern boundary of the Gulf Stream (also called the Northern Wall for its sharp temperature gradient) and TARA_146 was south of the southern boundary, in the subtropical gyre (Fig. 2 and Supplementary Figure S8).

**Figure 3:**
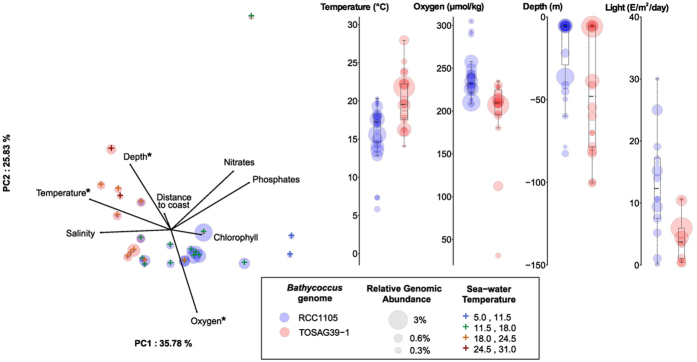
Relationships between environmental parameters and *Bathycoccus* genome abundance. Left: Principal component analysis. We only considered stations where we detected 98% of the genes for one *Bathycoccus* genome, and for which all environmental parameters were available (Oxygen, Nitrates, Phosphates, Chlorophyll, Sampling Depth, Water Temperature and Salinity). Crosses indicate stations, with a color scale corresponding to the water temperature. The distance to coast parameter corresponds to the shortest geographical distance to the coast. The two *Bathycoccus* are distributed along temperature and oxygen axes. Stars indicate parameters that statistically discriminate the two *Bathycoccus*. Right: Range of values of temperature, oxygen and sampling depth for parameters where a significant difference was detected between RCC1105 and TOSAG39-1.

Principal component analysis was used to assess the relationship between the genomic data and environmental parameters determined *in situ*^36^ complemented by satellite and climatology data (Supplementary Information). Temperature, oxygen, sampling depth and PAR (photosynthetic active radiation), though with less significant p-values for the latter, were related to the segregation of the two genomes (Fig. 3 and Supplementary Figure S9). The two *Bathycoccus* were found in temperature ranges from 0 to 32 °C and from 7 to 28 °C for RCC1105 and TOSAG39-1, respectively. On average, the TOSAG39-1 genome was found in waters 3 °C warmer than was RCC1105 (21.5 *vs.* 18.4 °C, p-value < 10^−3^, Fig. 3 and Supplementary Figure S10). Abundances were very low below 13 °C for both genomes, and above 22 °C for RCC1105. A similar discrimination was observed for oxygen: TOSAG39-1 was found in samples with lower oxygen content. For example, the TOSAG39-1 genome was abundant in the DCM of station 138 where O_2_ was low (31.2 μM, Fig. 3, Supplementary Figures S9 and S10), though no samples originated from anoxic waters^46^.

The two *Bathycoccus* were recovered from significantly different ranges of PAR, estimated from weekly averages of surface irradiance measurements extrapolated to depth using an attenuation coefficient derived from local surface chlorophyll concentrations^47^ (Fig. 3, Supplementary Figures S9 and S10, Supplementary Information). Both *Bathycoccus* could thrive in winter when the overall light availability is low (Supplementary Figure S8). Nutrient concentrations did not seem to explain the separation between the two *Bathycoccus*. We found RCC1105 in nutrient-rich surface waters and TOSAG39-1 mostly at the DCM in oligotrophic waters, close to the nutricline characterized by a significant upward flux of nutrients^48^,^49^. While RCC1105 was never abundant below 80 m, TOSAG39-1 extended down to almost 150 m (Fig. 3 and Supplementary Figure S10).

### Genomic plasticity

**Figure 4:**
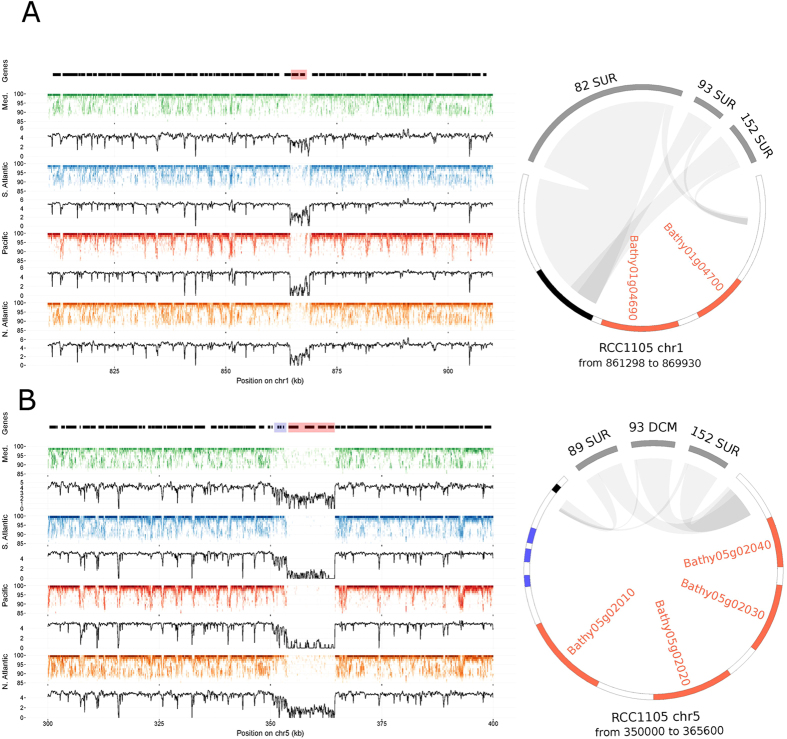
Evidence for cassettes of dispensable genes in *B. prasinos* RCC1105. Left and right sides of the figures represent fragment recruitment and genomic alignments of dispensable gene cassettes, respectively. Fragment recruitments plots are displayed by marine zones (left legend). Each dot corresponds to a given number of mapped reads at a given identity percent (indicated on the Y-axis). The density of mapped read is displayed as the black line plotted below each fragment recruitment plot. Gene positions are represented by black boxes on the top of the first fragment recruitment plot and dispensable genes are highlighted in red. Genomic alignments are represented as circos graphs^79^ on which dispensable genes are colored in red, and other genes are represented by black boxes. Left side and right side of the genomic region are connected to metagenomics contigs (gray segments), leaving in-between the locus of the dispensable gene cassette that remains unconnected to any metagenomic contig. Connections correspond to blast alignments positions. (**A**) 100- and 8.6-kb regions of chromosome 1 are represented on a fragment recruitment plot and on the circos graph, respectively. A two gene long cassette is represented. A massive decrease of read coverage appears on the fragment recruitment plot in all oceanic zones except in the Mediterranean Sea, which indicates that the two genes are present only in a sub-population in this basin. A similar pattern is observed in panel (**B**) for four consecutive genes for which fragment recruitment plots representing 100 kb of chromosome 5 suggest a presence in a Mediterranean sub-population and absence in other marine areas. The circos graph represents alignments along the 15.6-kb cassette locus with metagenomics contigs, which resulted in a gap that included three small genes (in blue) in addition to the four automatically detected dispensable genes. Fragment recruitment confirmed a significant, but not total, decrease of read coverage for these three genes in every oceanic zone, indicating that their presence or absence in the two sub-populations was widely distributed.

For each genome, we searched for evidence of gene gain or loss by analyzing gene content variations at the different stations. Lost or gained genes could be considered as dispensable genes or as present only in some genomic variants, therefore, characterizing a “pan-genome” analogous to what is observed in bacterial populations^50^. We analyzed the coverage of metagenomic reads that were specifically mapped at high stringency onto one genome and looked for traces of gene loss. To avoid false positives caused by conserved genes, we restricted this analysis to samples where 98% of the genes from one of the two *Bathycoccus* genome sequences were detected, and focused on genes that were detected in the metagenomes of at least four samples, and not detected in at least five samples. Metatranscriptomic data was used to select genes having an expression signal in at least six samples. Using these stringent criteria, we detected about one hundred dispensable genes for each genome (Supplementary Tables 1,4 and 5). Half of the RCC1105 dispensable genes (50/108) are located on chromosome 19, representing 70% of the genes on this chromosome. These genes have shorter coding and intronic regions than other genes (Supplementary Table 1), which is a property of the genes predicted on outlier chromosome 19^22^. Dispensable genes on regular chromosomes also tend to be shorter. Additionally, the distribution of dispensable genes on the genome is not random. Among the 72 genes of chromosome 19, 47 out the 50 dispensable genes are grouped into two long blocks at the chromosome end, leaving the first part of chromosome 19 almost free of dispensable genes (Supplementary Figure S11). Dispensable genes also appear clustered on regular chromosomes. Twenty-one out of 58 dispensable genes are in small cassettes, two to four gene-long, especially on chromosomes 2, 5 and 17 (Fig. 4 and Supplementary Figure S11). We verified the contiguity of the genomic regions around the dispensable genes by alignment with assemblies of metagenomics reads (Supplementary Information). We analyzed the pattern of loss of these dispensable cassettes in samples where they were not detected and obtained alignments that included gaps in place of dispensable genes (Fig. 4). Notably, cassette borders were at the same positions in the various samples, showing a low diversity at these loci. This suggests that a common or single breakpoint event occurred in the past. Fragment recruitments plots showed a homogenous decrease of read coverage along the contiguous dispensable genes, confirming that genomic losses or gains occurred at the scale of entire cassettes (Fig. 4 and Supplementary Figure S11). We examined the synteny between RCC1105 and TOSAG39-1 for the regions corresponding to the two cassettes illustrated in Fig. 4. We retrieved the orthologous genes situated around the cassettes in two TOSAG39-1 scaffolds in a clear syntenic relationship, but the cassettes genes were missing.

We observed an incomplete, but marked, depletion of read coverage for three contiguous genes on chromosome 5. These genes immediately precede the longest dispensable gene cassette. This incomplete read coverage depletion indicates that this genomic region only occurs in a sub-population, suggesting a sympatry or at least co-occurence of these two genomic forms. This pattern was observed in every oceanic basin (Fig. 4B) with the longest dispensable gene cassette spanning seven genes.

The function of these dispensable genes is unclear. Only 15 dispensable genes located on RCC1105 non-outlier chromosomes possess a protein Pfam domain (Supplementary Information, Supplementary Table 3). However, several of these genes might be involved in genomic rearrangements because they contain reverse transcriptase and HNH endonuclease domains and this could be linked to their dispensability. Intriguingly, the average relative transcriptomic activity is higher in dispensable genes than in non-dispensable genes (0.73 *vs.* 0.56, Mann-Whitney-Wilcoxon test p-value = 1.52E-4, Supplementary Table 1).

Beside these patterns suggesting gene gains or losses, we examined at a global level the genomic variation within populations of each *Bathycoccus*. This was done by fragment recruitment of the metagenomic reads of *Tara* Oceans samples onto the two reference assemblies. The distributions of nucleotide identities show a weak divergence between the reference assemblies and geographically distant samples, though higher for TOSAG39-1 than for RCC1105 (Supplementary Information, Supplementary Figure S12).

## Discussion

We provide a novel *Bathycoccus* genome assembly using a single-cell genomics approach. This assembly is estimated to be 64% complete, which is, to our knowledge, the most complete eukaryotic genome obtained to date by this approach. This relatively high level of completion was reached through the combination of several independent cells originating from the same population. It has been described that the enzymatic amplification of DNA which is inherent to single-cell genomics induces strong biases in sequencing depth along the genome, leading to partial and fragmented assemblies^51^. Here, this caveat appears reduced as the combined-SAGs assembly is significantly more complete than the assembly obtained from each of the individuals SAGs.

This *Bathycoccus* SAG assembly is significantly different from the previously described genome assembly, originating from the coastal Mediterranean strain RCC1105. The former corresponds to the B1 clade and the latter to the B2 clade as, defined recently^11^. Orthologous proteins of these two genomes share only 78% identity, which is similar to the 74% of amino-acid identity shared by the two sequenced *Ostreococcus* isolates which belong to different clades^52^.

A previous study^11^ estimated a lower genetic distance (82% of identical nucleotides) between the two *Bathycoccus* using metagenomic data. This difference is probably as expected because of the reduced dataset of highly conserved and single copy genes (1 104 genes) considered in the latter analysis. The evolutionary distance that separates the protein coding genes of these two *Bathycoccus* is slightly smaller than the one between two vertebrate lineages separated by more than 400 million years (mammal and fish share 72% of identity^53^) and larger than the one reported between many model organisms (for example, human and mouse share 85% of identity^54^,^55^). This high divergence in protein coding genes and the frequent genes rearrangement in chromosomes is hardly compatible with chromatid pairing required for intercrossing^56^ between the two *Bathycoccus*. Very few genes are highly conserved (>99% identity) between the two *Bathycoccus* and conserved genes are not clustered, which makes active genetic exchange by homologous recombination unlikely. Therefore, although the two *Bathycoccus* share 100% similar rRNA gene sequences, these genomic differences reflect two different, probably cryptic, species. Identical rRNA sequences have been previously reported in the yeast *Saccharomyces cerevisiae sensu stricto* clade^57^, or the haptophyte species *Emiliania huxleyi* and *Gephyrocapsa oceanica,* which also have identical 18S rRNA gene sequences, but quite different morphologies^58^.

The combination of genomics and environmental data from a large set of oceanic samples revealed the distinct ecological preferences of the two *Bathycoccus* with respect to depth, temperature, light and oxygen. TOSAG39-1 is usually found in warmer but deeper and darker water than RCC1105. TOSAG39-1 seems to be well adapted to the DCM conditions, which would explain its presence in oligotrophic marine zones where nutrients are found deeper.

Numerous marine bacteria show geographical variation of their gene repertoire^59^,^60^,^61^,^62^,^63^ which affects genomic regions that generally represent only a few percent of the total genome^61^ and has been proposed, in some cases, to result from horizontal transfer. In *Prochlorococcus*, genomic islands are thought to be related to niche adaptation^63^ because they host ecologically important genes^60^. A comparison of two *Prochlorococcus* ecotypes revealed that differences in gene content were related to high-light *vs.* low-light adaptation^64^. Such adaptations have been hypothesized in species closely related to *Bathycoccus*, like *Ostreococcus*^17^, but are still a matter of debate^9^. Our data show that the depth and light ranges of the two *Bathycoccus* are different but overlapping, with TOSAG39-1 extending deeper. Interestingly, the surface samples where TOSAG39-1 was detected correspond to sites that undergo vertical mixing (Aghulas and Gulf Stream). Temperature also seemed to influence the distribution of the two *Bathycoccus*, as for example along the Gulf Stream where one type is more prevalent on the West side and is replaced by the other type eastward as water cools down. Among eukaryotes, several examples of correspondence between temperature and geographical distribution have been reported, such as for the heterotrophic MAST-4^26^,^65^ and the Arctic ecotype of *Micromonas*^8^. TOSAG39-1 was also observed at low O_2_ concentrations at Costa Rica Dome station 138, an area of high biological production in the East equatorial Pacific^66^ where picoplankton can be very abundant^67^. This could reflect the fact that since TOSAG39-1 is better adapted to low light conditions it could be found deeper in the water column where suboxic conditions are developing, rather than having a specific capacity to withstand low O_2_.

The wide geographical distribution and relatively high abundance of *Bathycoccus* observed here implies a capability to thrive across a range of ecological niches. Dispensable genes could correspond to the genomic traces of this adaptation. Intriguingly, dispensable *Bathycoccus* genes have genomic features similar to those of chromosome 19 genes, such as a lower GC content. This suggests that these genes may have been located on chromosome 19 ancestrally and have undergone subsequently inter-chromosomal translocations. A recent experimental evolution experiment of *Ostreococcus tauri* inoculated with a large quantity of virus, Otv5, provided evidence that genes on outlier chromosome 19 are up-regulated in viral-resistant cell lines and that the size of this chromosome varies in resistant lines^68^. Our results on gene content plasticity in Chromosome 19 is consistent with the immunity chromosome hypothesis: frequent events of gene birth and gene loss may thus be the genomic traces of a microalgal – virus evolutionary arm race.

Dispensable genes possess features of so-called *de novo* genes, genes emerging from previously noncoding regions. These genes are an important class of unknown genes and challenge evolutionary sciences^69^,^70^. It has been hypothesized that cosmopolitan bacteria would hold specific genes or gene variants due to their ecological properties^71^. Cosmopolitan marine lineages are exposed to a range of contrasted environmental constraints, raising the question of their genomic plasticity. The high turnover of a certain class of genes restricted to some environmental conditions might be an evolutionary advantage for rapid acclimation related to being cosmopolitan.

The amplification biases inherent to the Single Cell Genomics approach do not in general allow recovering full genomes from environmental protists. However even incomplete SAG assemblies are sufficient to allow mapping of environmental metagenomes and to determine the distribution of genotypes that are not resolved by traditional marker genes or metabarcodes. In the case of *Bathycoccus* we provide the distribution of two clades, corresponding to the genomes of RCC1105 (clade B1) and to the genome of TOSAG39-1 (clade B2) and identify environmental parameters underlying these distributions. Our observations unfortunately do not cover all oceanic ecosystems, particularly the polar zones. Future analysis of additional genomes and transcriptomes of wild and cultured *Bathycoccus* will improve the accuracy of the environmental niches of the two types of *Bathycoccus*.

## Material and Methods

During the *Tara* Oceans expedition^34^,^35^, we collected and cryo-preserved samples at station TARA_039 situated in the Arabian Sea (Supplementary Figure S13, oceanographic conditions are available in reference^36^). In the laboratory, single cells were sorted by flow cytometry based on their size and chlorophyll autofluorescence. Four *Bathycoccus* cells were identified following DNA amplification and 18 S rDNA sequencing^37^. The four amplified genomes (A, B, C, D - Table 1) were individually sequenced using Illumina HiSeq technology, and a suite of tools was used to obtain single-cell final assembly (Supplementary Information). Firstly, individual assemblies were generated using a colored de Bruijn graph-based method^72^ and then a final assembly, named here as TOSAG39-1, was generated comprising gap-reduced scaffolded contigs, using SPAdes, SSPACE and GapCloser^73^,^74^,^75^ (Supplementary Figure S14). The four cells had identical 18 S sequences and came from the same 4 mL sample, so it is reasonable to presume they were of the same population.

Quality control filters detected and removed contigs or scaffolds that did not correspond to *Bathycoccus* nuclear DNA (Supplementary Figure S14, Supplementary Information). Direct comparisons of sequence assemblies detected putative DNA contamination from other SAGs that were sequenced in the same laboratory and scaffolds corresponding to organelles.

We predicted exon-intron gene structures by integrating various coding regions data. We aligned the reference protein set of the published *Bathycoccus* RCC1105 genome^22^ to our assembly. We extracted and sequenced polyA mRNA from *Tara* Oceans samples. We aligned this eukaryote metatranscriptome on TOSAG39-1 assembly. We also used a public protein databank^76^ and the Marine Microbial Eukaryote Transcriptome Sequencing Project (MMETSP) collection of marine protist transcriptomes^42^. In addition, we performed direct *ab initio* prediction by calibrating and running the Markov model implemented in snap^77^. Integrating and combining all this evidence provided a final set of genes, using a process based on Gmorse software rationale^78^. We evaluated the relative genomic abundance of each genome for two sampled depths (surface and DCM) at the 76 *Tara* Oceans stations (122 samples in total, Supplementary Figure S13) by recruiting metagenomic reads^24^. We mapped metagenomic reads directly from 0.8–5 μm organism-size fraction samples onto genome assemblies, and estimated the relative contribution of each *Bathycoccus* genome in the metagenomes. To obtain a proper genome abundance estimate, we developed methods to select genome-specific signals only (Supplementary Information). We discarded highly conserved genes that were detected by direct sequence comparisons.

A more detailed description of methods is available in the online supplementary information.

## Additional Information

**Accession codes:** This article is contribution number 48 of Tara Oceans. Physicochemical parameters from all Tara Oceans samples are available at Pangea (http://doi.pangaea.de/10.1594/PANGAEA.840721); metagenomics reads can be downloaded at SRA under identification study number PRJEB402 (https://www.ebi.ac.uk/ena/data/view/PRJEB402). The sequences of TOSAG39-1 were deposited and are available at EMBL/DDBL/GenBank under accession number ERA768231.

**How to cite this article**: Vannier, T. *et al*. Survey of the green picoalga *Bathycoccus* genomes in the global ocean. *Sci. Rep.* **6**, 37900; doi: 10.1038/srep37900 (2016).

**Publisher's note:** Springer Nature remains neutral with regard to jurisdictional claims in published maps and institutional affiliations.

## Supplementary Material

Supplementary Information

## Figures and Tables

**Table 1:** Assembly summaries of TOSAG39-1.

SAG Assembly	Total Size (Mb)	N50 (kb)	NG50^1^ (kb)	Genome Completion (%)
A	3.5	14.8	NA	30.8
B	4.7	14.5	NA	27.7
C	3.7	24.1	NA	21.5
D	4.1	18.1	NA	26.0
(A) + (B) + (C) + (D)^2^	8.0	16.6	0.9	44.6
Combined ABCD^3^	10.1	14.1	6.0	64.0

^1^The longest assembly contigs covering together half of the genome size (15 Mbp) are each longer than the NG50. This evaluation was not possible for the four individual cell assemblies for which the total assembly sizes are shorter than half of the genome size.

^2^A + B + C + D corresponds to a non-redundant merging of contigs from individual assemblies.

^3^Combined ABCD corresponds to the co-assembly process.
