# Supplementary material for: Survey of the green picoalga Bathycoccus genomes in the global ocean
Source: Sci Rep. 2016 Nov 30;6:37900. doi: 10.1038/srep37900 (PMC5128809; doi:10.1038/srep37900)
Supplement: Supplementary Information [file srep37900-s1.pdf]

Supplementary Information for

Survey of the green picoalga *Bathycoccus* genomes in the global ocean

Thomas Vannier[1,2,3], Jade Leconte[1,2,3], Yoann Seeleuthner[1,2,3], Samuel Mondy[1,2,3], Eric Pelletier[1,2,3], Jean-Marc Aury[1], Colomban de Vargas[4], Michael Sieracki[5], Daniele Iudicone[6], Daniel Vaulot[4], Patrick Wincker*[1,2,3] & Olivier Jaillon*[1,2,3]

[1]CEA - Institut de Génomique, GENOSCOPE, 2 rue Gaston Crémieux, 91057 Evry France.

[2]CNRS, UMR 8030, CP5706, Evry France.

[3]Université d'Evry, UMR 8030, CP5706, Evry France.

[4]Sorbonne Universités, UPMC Université Paris 06, CNRS, UMR7144, Station Biologique de Roscoff, 29680 Roscoff, France.

[5]National Science Foundation, 4201 Wilson Blvd., Arlington, VA 22230, USA.

[6]Stazione Zoologica Anton Dohrn, Villa Comunale, 80121 Naples, Italy

*Correspondence: Olivier Jaillon (ojaillon@genoscope.cns.fr) and Patrick Wincker (pwincker@genoscope.cns.fr).

Genomic data

Bathycoccus RCC1105[1] was isolated in the bay of Banyuls-sur-mer at the SOLA station at a depth of 3 m in January 2006. Sequences were downloaded from the Online Resource for Community Annotation of Eukaryotes[2]. Two metagenomes of uncultured *Bathycoccus* sorted by flow cytometry[3] were obtained from samples taken in the Eastern South Pacific Ocean at depths of 5 and 30 m (33°59′46″S, 73°22′10″W and 33°51′37″S, 73°20′24″W). Their accession numbers are CAFX01000000 and CAFY01000000. A third flow cytometry sorted metagenome[4] originated from the Deep Chlorophyll Maximum layer (DCM) at station OLIGO in the Atlantic Ocean (12°22′40″N, 27°14′27″W) with accession number AFUW01000000.

Single-cell isolation and amplification

The four cells composing the final genome sequence assembly of TOSAG39-1 (for *Tara* Oceans Single Amplified Genome from Station 39 numbered 1) originated from a sample of the *Tara* Oceans expedition, obtained in December 2009 in the Arabian Sea (18°34′52.3″N, 66°33′43.7″E) at station TARA_039 in surface (Supplementary Figure S13). Samples were preserved in 6% glycine betaine final and frozen quickly in liquid nitrogen. Samples were shipped to the Bigelow Laboratory Single Cell Genomics Center where they were thawed. Single cells were sorted into a lysis buffer by flow cytometry based on their cell size and chlorophyll content. The DNA content of each cell was amplified separately using Multiple Displacement Amplification (MDA), following previously described protocols[5]. The identification of cells was based on the 18S rRNA gene sequence. After multiple alignments using MUSCLE[6], it appeared that the 18S rRNA sequence of TOSAG39-1 was strictly identical to that of *Bathycoccus prasinos* (GenBank: AY425315, FN562453).

DNA sequencing and assembly

The four cells, A, B, C and D were sequenced independently on 1⁄8th Illumina HiSeq lane, producing a total of 96 million 101-bp paired-end reads. For the combined-SAG assembly, we pooled the reads from

the different cells to increase the completion of the final assembly. To ensure that genomes of these cells could be correctly co-assembled, we first analyzed the contribution of each cell to a global assembly using the HyDA assembler[7]. HyDA produced a colored de Bruijn graph in which most contigs were covered by reads from at least three different cells, suggesting that the genomes were close enough to be successfully co-assembled. We used SPAdes 2.4[8] using parameter k = 21, 33 and 55 to obtain the final assembly, and we scaffolded contigs using the SSPACE program[9]. We used GapCloser (v 1.12-6 from SOAPdeNovo2 package[10]) with default settings to perform gap filling on the resulting scaffolds. Scaffolds shorter than 500 bp were discarded from the assembly.

We obtained individual assemblies for each cell, A, B, C and D separately using the same versions of SPAdes, SSPace and GapCloser. We computed a merged-assembly by pooling all scaffolds from the four individual assemblies and removing the redundancy using CD-HIT[11,12] v 4.6.1. Scaffolds with $\geq$ 95% identity and $\geq$ 80% overlapping (considering the shortest sequence) were clustered together and the longest scaffold of each cluster was kept as representative. The combined-SAGs assembly is the longest and appears as the most complete (Table 1).

Gene prediction on the TOSAG39-1 assembly

To predict different structures or specific genes that would be absent from the RCC1105 genome, we performed a *de novo* gene prediction using three different resources: protein mapping from a custom database enriched in marine protists transcripts, including the RCC1105 proteome; *ab initio* gene predictions; and transcriptional evidence from *Tara* Oceans metatranscriptomic data. Before this process, we masked the TOSAG39-1 assembly against repeated sequences using RepeatMasker version open-3.3.0[13].

We then mapped all proteins with BLAST+ 2.2.27[14] (e-value < 10^{-2}). The reference database was built with Uniref100[15] (version July 25th 2013) and the MMETSP transcriptomes[16] (version August 2013). We obtained a total of 6 560 distinct matches. For *ab initio* predictions, we used the SNAP predictor[17] after

calibration on *Bathycoccus prasinos* RCC1105 gene models. This resulted in the prediction of 6 797 gene models. Biological evidence was also provided by *Tara* Oceans metatranscriptomes. After mapping metatranscriptomic reads from all *Tara* Oceans samples of the 0.8-5 µm size fraction, we used the Gmorse pipeline[18] to define the gene structures from vertical coverage. We applied a minimum read coverage threshold of 32 because of the large abundance of *Bathycoccus* in *Tara* Ocean samples. We detected 6 112 genes. We finally integrated protein mapping, SNAP *ab initio* predictions and metatranscriptome derived gene models using a combiner process modified from the Gmorse software[16] and obtained 6 444 gene models. Further quality control filtering on putative non-*Bathycoccus* nuclear DNA reduced the final gene set to 6 157 (see below). Comparisons of TOSAG39-1 and RCC1105 gene sets are given in Supplementary Table 1.

TOSAG39-1 and RCC1105 genomic comparison

Best reciprocal hits (BRH)

We identified orthologous genes between RCC1105 and TOSAG39-1. We aligned each pair of genes using the Smith-Waterman algorithm[19] and retained alignments having a score higher than 300 (BLOSUM62, gapo = 10, gape = 1). We defined 4 153 best reciprocal hits as orthologs. The distribution of the percent identities for these BRH between the two *Bathycoccus* genomes is shown in Supplementary Figure S3.

Synteny and collinear genes analysis

We aligned the RCC1105 genomic data against the twenty longest TOSAG39-1 scaffolds (containing 656 genes) using promer (default parameter) from the MUMmer 3.19 package[20]. We used mummerplot to select RCC1105 chromosomes that corresponded to TOSAG39-1 scaffolds. We identified 18 scaffolds having an alignment covering their entire length with 11 chromosomes. We identified 573 RCC1105 genes localized within these syntenic regions. One of the two remaining scaffolds had matches with one RCC1105 contig that is not mapped to any chromosome, and the other could not be aligned and had a

lower GC% (0.44 *vs*. 0.48 averages for the other scaffolds) suggesting a chromosome 19 origin. To identify genes that are shared between the two genomes, we compared TOSAG39-1 scaffolds and RCC1105 in the six translated frames using tblastx[14] (e-value < 10^{-3}). We visually inspected genomic alignment regions using Artemis[21] and identified 52 RCC1105 genes localized in syntenic regions that lacked any alignments. We further compared these 52 genes against the whole genome at the protein level with tblastx[14] (e-value < 10^{-3}) and identified a total of 24 exclusive genes.

Comparison between *Bathycoccus* genomes and MMETSP transcriptome

We compared the RCC1105 and TOSAG39-1 gene sets to the two *Bathycoccus* transcriptomes available in the MMETSP collection[16]. We computed the best reciprocal hit at the amino acid level, as defined previously, and distributed their percentage of identity. We identified unambiguously MMETSP1460 (culture strain RCC716) and MMETSP1399 (culture strain CCMP1898) as corresponding to TOSAG39-1 and RCC1105, respectively (Supplementary Figure S5)

Comparison between *Bathycoccus* genome assemblies and metagenomes containing *Bathycoccus*

We compared by tblastn[14] (selecting e-value lower than 10^{-3}) the gene sets of RCC1105 and TOSAG39-1 to the two metagenomes (T142 and T149) from the Chile upwelling[3] and to the metagenome from the Atlantic Ocean DCM[4,22]. We selected matches covering more than 80% of the genes. We identified that RCC1105 corresponds to the T142 and T149 metagenome and TOSAG39-1 corresponds to the Atlantic Ocean metagenome (Supplementary Figure S5).

Metagenomic fragment recruitment

In order to analyze the diversity of *Bathycoccus* genomes and of dispensable genes, metagenomic reads from the *Tara* Oceans 0.8–5-µm fraction samples were recruited to whole sequence assemblies. We used Bowtie2-2.1.0[23] to align reads longer than 80 bp. We retained matches having more than 80% identity and more than 30% of high-complexity bases. From the initial 122 samples, we further analyzed the 36

samples for which at least 98% of the genes of *Bathycoccus* were detected (more than one mapped read). Using R-package ′ggplot2′[24], we displayed the density of reads mapping along the genome in 5 000-bp bins and 1% identity height (Supplementary Figure S11). This representation reduces the granularity of the Y-axis, particularly for high identity levels, caused by the short length of reads.

Gene set filtering

Mitochondrial and plastid genes

tblastn (e-value < 10^{-20})[14] was used to compare the mitochondrial and chloroplast RCC1105 proteins against TOSAG39-1 scaffolds. To check the validity of these scaffolds, we compared these selected scaffold against the nr database[25] using blastn[14]. We identified 35 genes as putatively of chloroplast or mitochondrial origin. The corresponding scaffolds were not further considered in the analysis.

Foreign sequences in TOSAG39-1 assembly

To improve detection of non-*Bathycoccus* DNA sequences in the TOSAG39-1 assembly, we used the results of metagenomic fragment recruitments for *Tara* Oceans samples. We postulated that assembly contigs corresponding to *Bathycoccus vs.* to non-*Bathycoccus* would be mapped by metagenomic reads at different coverages in the various samples. Therefore, we analyzed the variations of coverage of each gene along *Tara* Oceans samples to retrieve the specific *Bathycoccus* coverage profile. We assumed that the coverage profile of the majority of genes was the signature of TOSAG39-1. Considering these profiles as a time series, we used the ”diss.CORT“ function of the ”TSclust“ R-package[26] to compute distances based on abundance values and spatial correlation between profiles. We tagged 533 genes having a profile quite different from that of TOSAG39-1. However, we untagged from this list genes having an ortholog in *Bathycoccus prasinos* RCC1105. Finally, we discarded scaffolds containing tagged genes only. The aim of this filter is to discard the maximum of contigs that have an outlier statistical signal on fragment recruitment to avoid any putative bias due to atypical genomic region. Using this approach, we removed 223 scaffolds from the assembly. We compared these scaffolds on public databases using blast[14]. Due to

the stringency of this filter, some of these scaffolds (37.8%) seem to correspond to *Bathycoccus*, but the majority doesn't have any match or match different other organisms (Supplementary Table 6).

We also followed this rationale to detect genes having "outlier" profiles. We identified 826 and 1 051 genes on RCC1105 and TOSAG39-1, respectively. Among these, 111 and 223 were identified as cross-mapped genes (see below).

Estimation of cross-species mapped genes

In order to analyze the abundance of the two *Bathycoccus* genomes in the *Tara* Oceans metagenomic samples, we checked the possibility that some genes could be cross-mapped, that is genes that could be mapped by metagenomic reads from both genotypes. These genes could lead to a background signal in species detection survey. We identified 1 057 and 1 020 genes from TOSAG39-1 and RCC1105, respectively, that could be aligned on the other genome using Bowtie[23]. In order to do this, we fragmented one genome into 100-bp fragments that we mapped on the second genome to simulate metagenomics fragment recruitment conditions. We retained results having more than 95% identity. Since TOSAG39-1 is 64% complete, we extrapolated the total number of cross-mapped genes to about 1 500.

Abundance counts

Relative genomic abundance

We mapped metagenomic reads on RCC1105 and TOSAG39-1 genome sequence using Bowtie2 2.1.0 aligner with default parameters[23]. We filtered out alignments corresponding to low complexity regions using the dust algorithm[27] and we discarded alignments with less than 95% mean identity or with less than 30% of high complexity bases. For each *Bathycoccus,* we computed relative genomic abundances as the number of reads mapped onto non-outlier genes normalized by the total number of reads sequenced for each sample. We took into account the estimated fraction of genome recovery of TOSAG39-1 assembly to extrapolate the number of reads mapped on non-outlier genes to a complete genome assembly. Cross mapped genes, organelles and outlier genes were dismissed for the calculation. We generated the world

maps and heatmaps with R-package maps_2.1-6, mapproj_1.1-8.3, gplots_2.8.0 and mapplots_1.4 (version R-2.13, https://cran.r-project.org/web/packages/maps/index.html).

$RPKM_{MG}$ and $RPKM_{MT}$

Metagenomic and metatranscriptomic read counts per gene ($RPKM_{MG}$ and $RPKM_{MT}$) correspond to the number of mapped reads per gene (intron plus exon for $RPKM_{MG}$) or per CDS (for $RPKM_{MT}$) divided by the total number of reads sequenced for each sample multiplied by gene length. We used the following formula for figures: $\frac{\log(1+(RPKM*10^9))}{\log(2)}$. We investigated relative transcriptomic activity of genes by dividing $RPKM_{MT}$ by $RPKM_{MG}$. If $RPKM_{MT} > 0$ but $RPKM_{MG}$ is null, we used the median of the total $RPKM_{MG}$.

Metabarcoding

Metabarcoding abundance values (V9 region of 18S rRNA genes) were extracted from a previous study[28] and correspond to the proportion of all eukaryotic reads assigned to *Bathycoccus.*

Analyses of dispensable genes

Identification and characterization

To detect variations in gene content of the two *Bathycoccus* genomes in the different samples, in particular gene loss, we analyzed the coverage of metagenomic reads that were specifically mapped on each genome at high stringency. To avoid putative background signals, we restricted this analysis to samples where 98% of the genes were detected (metagenomic abundance > 0). We retained 34 samples for RCC1105 and 21 samples for TOSAG39-1. We then focused on genes that were detected in at least four samples, and not detected in at least five samples. We obtained 108 and 106 dispensable genes in RCC1105 and TOSAG39-1, respectively. We performed a Mann-Whitney-Wilcoxon test (using R function wilcox.test

with default parameters) to compare RPKM values and gene length between dispensable and non-dispensable genes. We considered a significant difference at a p-value < 10^{-3}.

Validation of dispensable cassette genes in metagenomes

We aimed to validate the genomic pattern of gain or loss of cassettes of dispensable genes on RCC1105 using long metagenomic contigs from the *Tara* Ocean expedition data. We selected *Tara* Oceans stations having a high abundance of RCC1105 and a negligible abundance of TOSAG39-1 (relative abundance < 0.05%). We assembled merged metagenomic reads using SOAPdenovo[10] and a kmer size of 31. Most of the metagenomics contigs were short (N50 sizes ranged from 804 to 836 nt in the different samples) because of the difficulty of assembling eukaryotic metagenomes. However, we identified by blastn[14] several long metagenomics contigs that covered two dispensable cassettes, including the longest one. These metagenomics contigs were from the following stations and depths: TARA_082 surface, TARA_093 surface, TARA_152 surface, TARA_089 surface, TARA_093 DCM and TARA_152 surface (Figure 4, Supplementary Figure 13). These alignments confirmed the total absence of these dispensable cassettes in these metagenomic contigs. Furthermore, the positions of the insertion or deletion of a given cassette were identical for several metagenomic contigs, indicating a common event and suggesting the existence of only two genomic forms at these genomic positions in these samples

Analysis of environmental parameters

We used physicochemical parameter values related to the expedition sampling sites and available in the Pangaea database[29]. We extrapolated PAR values (corresponding to weekly averages values of Photosynthetically Active Radiation) at sample depth using the following formula with *k* derived from surface chlorophyll concentration (Chl_{sur}) using the following published formulas[30].

$$PAR(Z) = PAR(0) * \exp(-k * z)$$

$$x = \log(Chl)$$

$$log(Z) = 1.524 - 0.426x - 0.0145x^2 + 0.0186x^3$$

$$k = \frac{-\ln(0.01)}{Z}$$

PAR values were only available for 59 out of 122 samples among which 21 out of the 36 samples contained abundant *Bathycoccus* genome. Consequently PAR was not included into the principal component analysis presented in figure 3, as it would have reduced the data set considerably. A principal component analysis including PAR values is presented in Supplementary Figure S9 and did not alter our conclusions.

We carried these analyses for stations for which at least 98% of genes from one of the two *Bathycoccus* were detected. For each parameter, we performed a Mann-Whitney-Wilcoxon test (using the R function wilcox.test with default parameters) between the TOSAG39-1 and RCC1105 sets of values.

rRNA operon comparison

The *Bathycoccus* RCC1105 rRNA operon was used as the reference sequence to align the rRNA operons of TOSAG39-1, of two metagenomes (T142 and T149) from the Chile upwelling[3], of a metagenome from the Atlantic Ocean DCM[4,22], and the ITS from strains RCC715 and 716 (Genbank accession KT809427, KT809428) that have been isolated from the Indian Ocean. The alignments were done with MAFFT, as implemented in Geneious 7.1 (http://www.geneious.com/).

Functional analysis of dispensable genes

We predicted functional annotations of protein domains using CDD database (version v3.11)[31].

Supplementary Figures

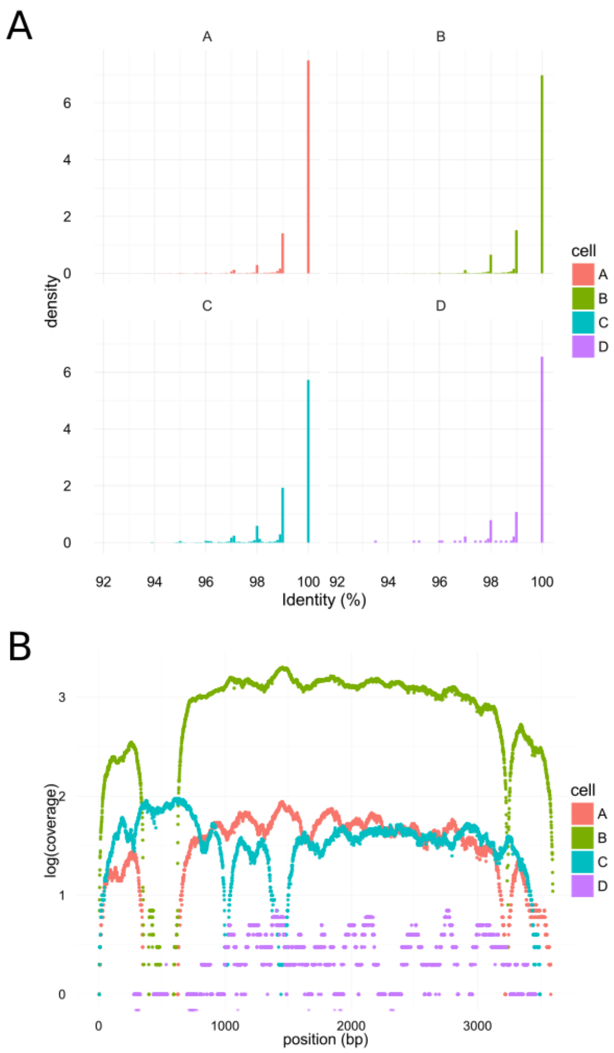

Figure S1. A. Distribution of identity percent of reads from each individual cell A (red), B (green), C (blue) and D (purple) once mapped onto the final combined SAG assembly. B. Example of the contributions of reads of each cell A (red), B (green), C (blue) and D (purple) along one contig of the final combined SAG assembly. X axis correspond to position and Y axis to coverage (log scale).

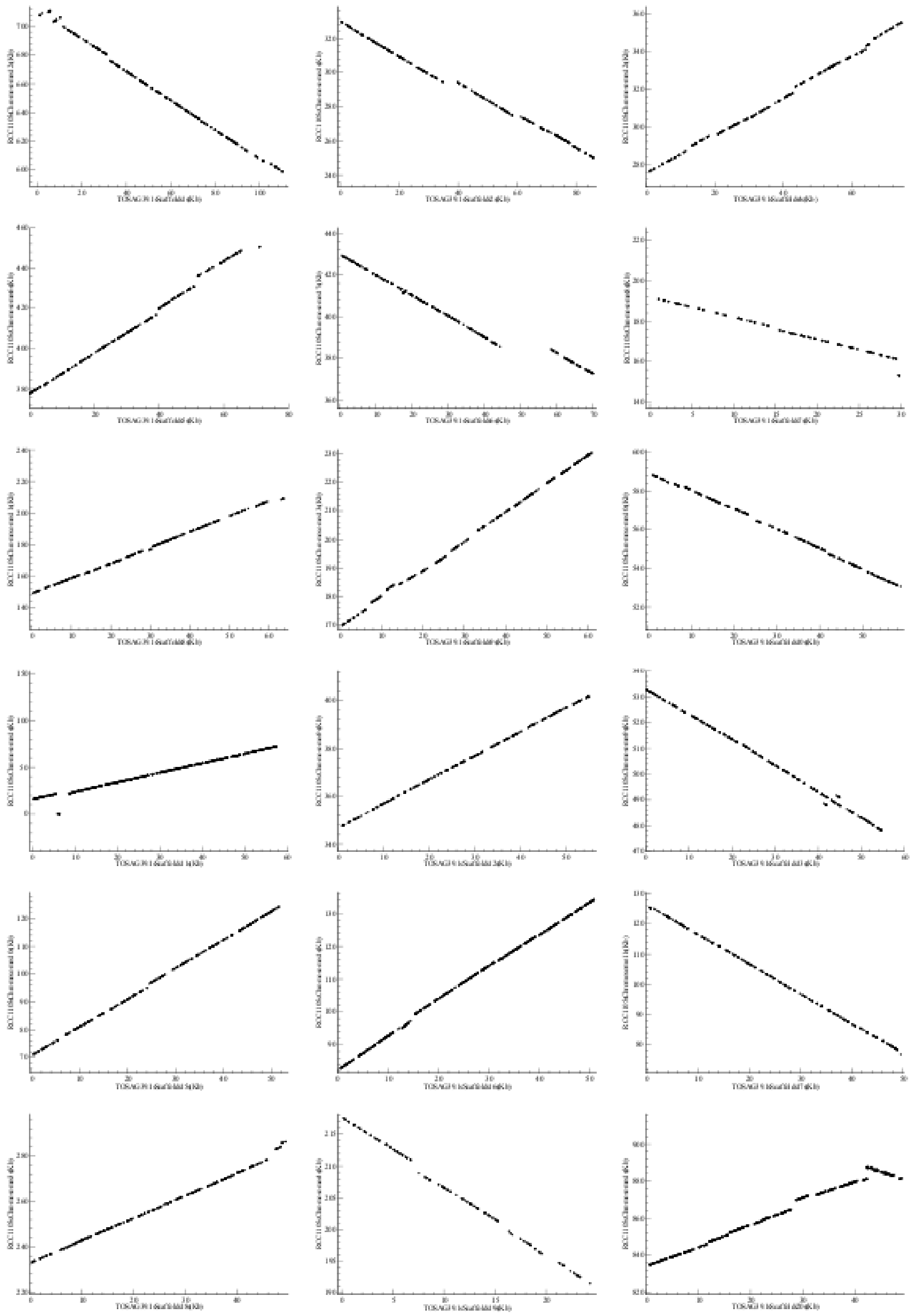

Figure S2. Synteny conservation between the two *B. prasinos* genomes. The RCC and TOSAG39-1 genomes are displayed on the X- and Y-axis, respectively. Dots correspond to regions conserved at the protein level (tblastx hits). Only the 18 longest scaffolds of TOSAG39-1 are represented. The two genomes are largely collinear and present only local and small rearrangements.

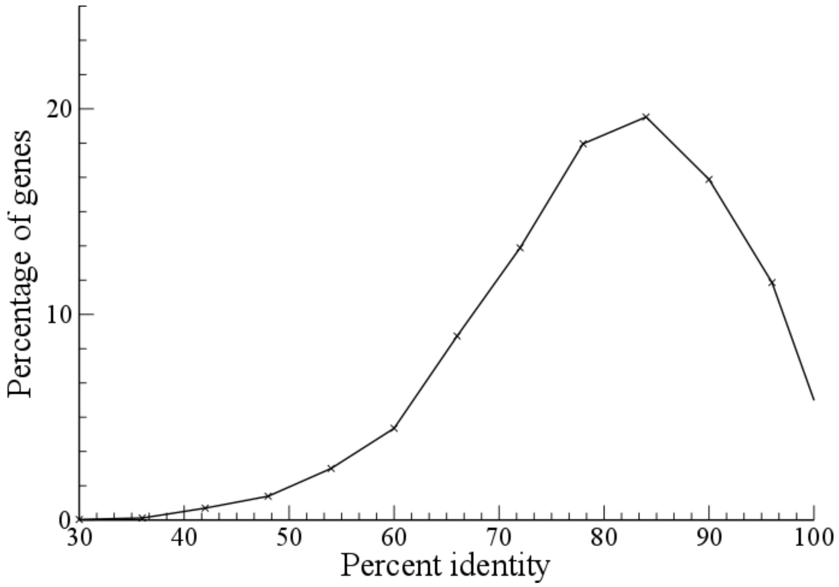

Figure S3. Distribution of orthologous gene divergence at the protein level between *Bathycoccus* RCC1105 and TOSAG39-1.

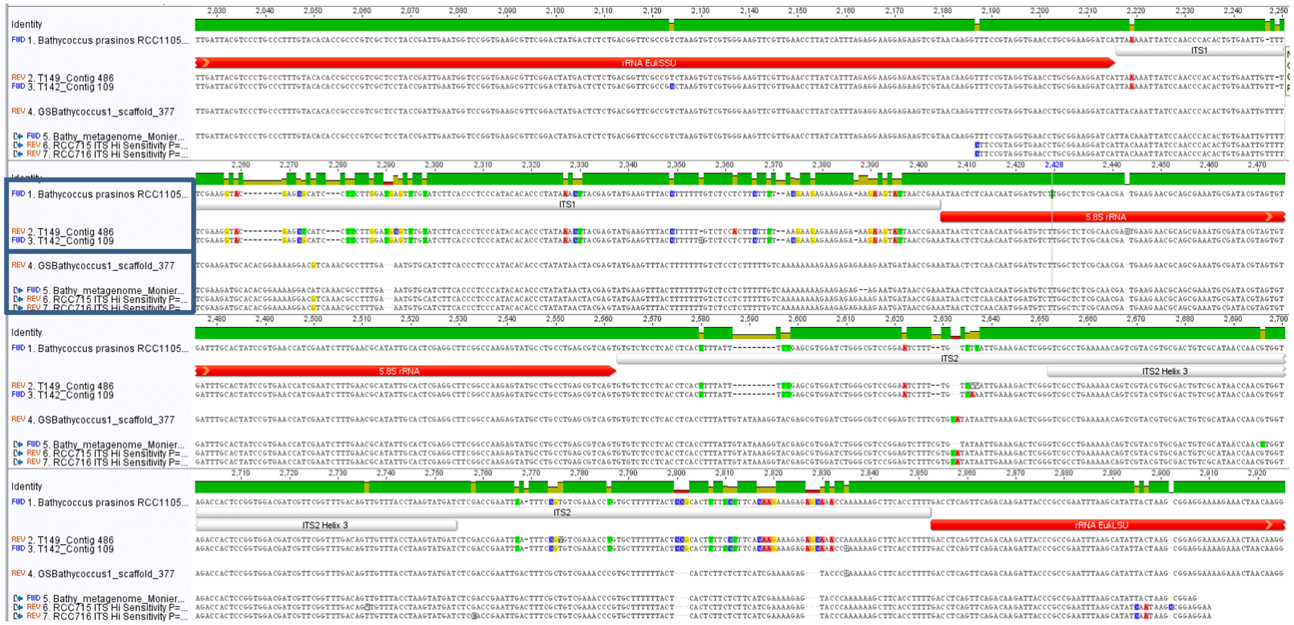

Figure S4. rRNA operon. Comparison of the rRNA ITS region between the two *Bathycoccus* genomes. RCC1105 and two metagenomes from the Chile upwelling[3] share identical ITS1 and ITS2, while TOSAG39-1 ITSs are identical to those of a metagenome from the Atlantic Ocean DCM[4] and to those from strains RCC715 and 716 that were isolated from the Indian Ocean.

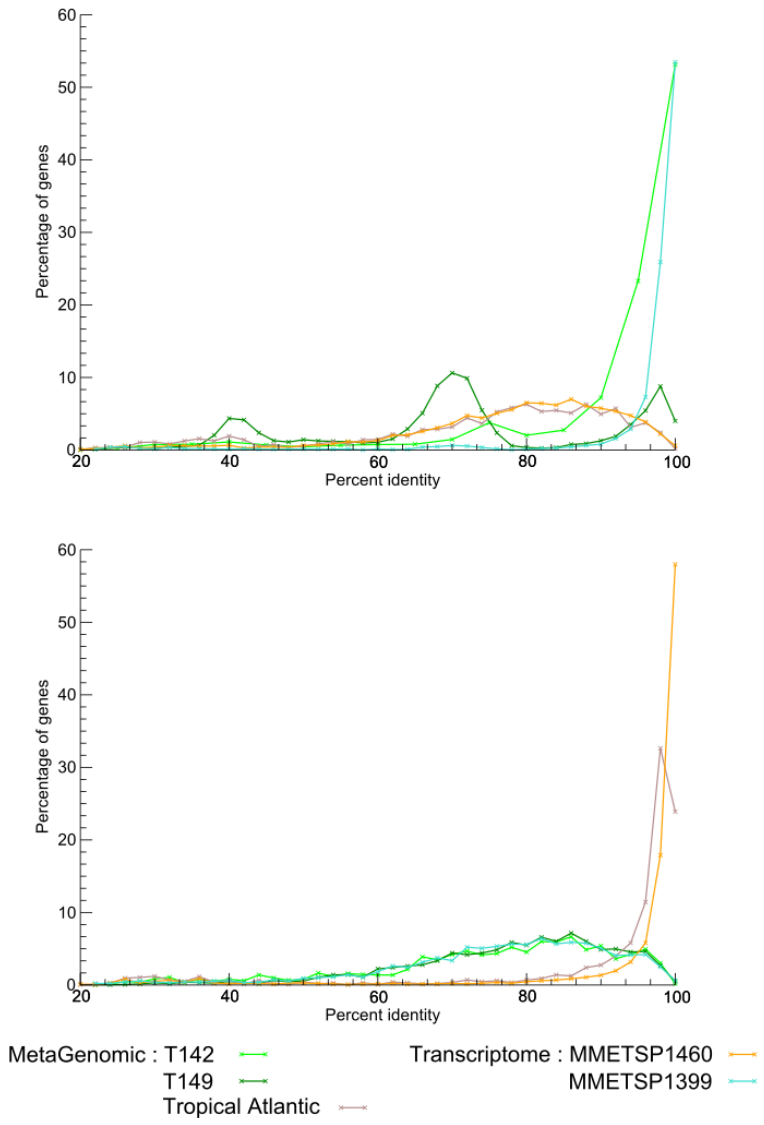

Figure S5. Affiliations of three metagenomes containing *Bathycoccus* and two *Bathycoccus* transcriptomes of the MMETSP database to the two genome assemblies. Distributions correspond to similarities at the amino acid level for one *Bathycoccus* genome assembly (top: RCC1105, bottom: TOSAG39-1) with two *Bathycoccus* transcriptomes (MMETSP1460 and MMETSP1399) and with three metagenomes containing *Bathycoccus*. MMETSP1399 transcriptome and T42 and T149 metagenomes correspond to RCC1105 genome, whereas MMETSP1460 and the tropical Atlantic metagenome correspond to TOSAG39-1.

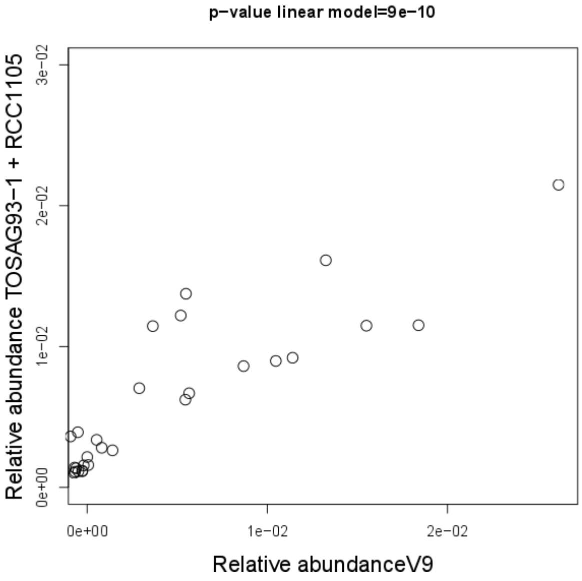

Figure S6. Correlation between the abundance of *Bathycoccus* estimated from whole metagenomes (two genomes summed) and V9 amplicons abundances.

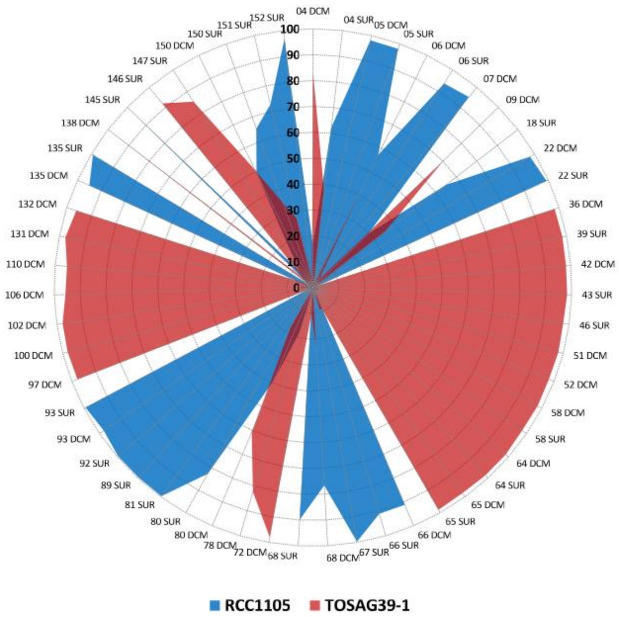

Figure S7. Relative contribution of each genome at *Bathycoccus*-rich stations. Within the 58 DCM and surface samples where *Bathycoccus* metagenomic abundance represents more than 0.01%, one of the two *Bathycoccus* genome was dominant (>70% of the *Bathycoccus* metagenomic reads) in 91% of the cases. The two genomes were measured in similar proportion (range 40% – 60%) in only two samples (stations 6 and 150 at the DCM).

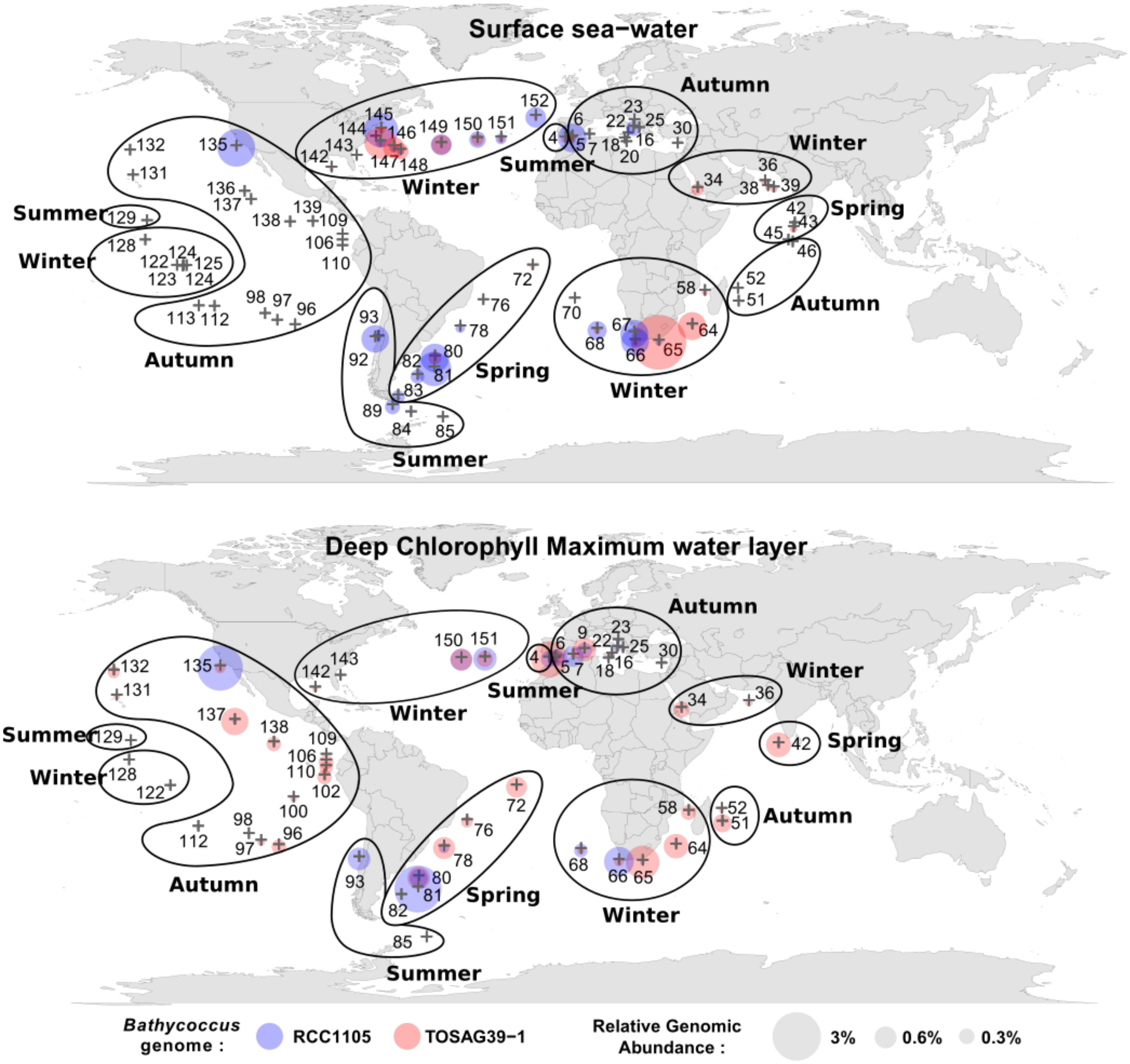

Figure S8. Map of relative metagenomic abundances of the two *Bathycoccus* in *Tara* Oceans stations with sampling season. This map was created using R-package maps_2.1-6, mapproj_1.1-8.3, gplots_2.8.0 and mapplots_1.4 (version R-2.13, https://cran.r-project.org/web/packages/maps/index.html).

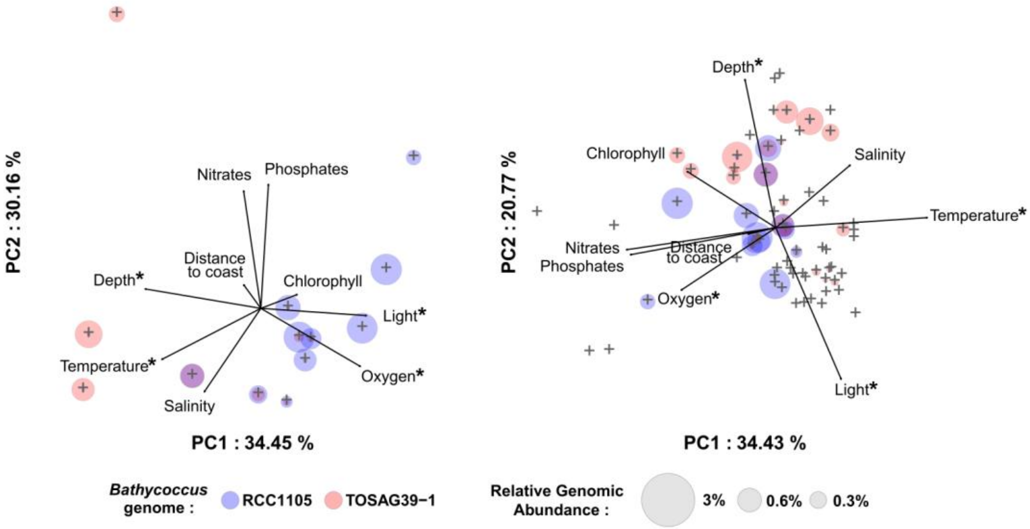

Figure S9. Principal Component Analysis including Photosynthetically Active Radiation (PAR). Left: Using only 13 samples for which we measured a large relative genomic abundance of *Bathycoccus* that have available PAR (indicated as light). Right: Idem but with all *Tara* Oceans samples that have available PAR values (indicated as light). Stars indicate parameters statistically discriminant.

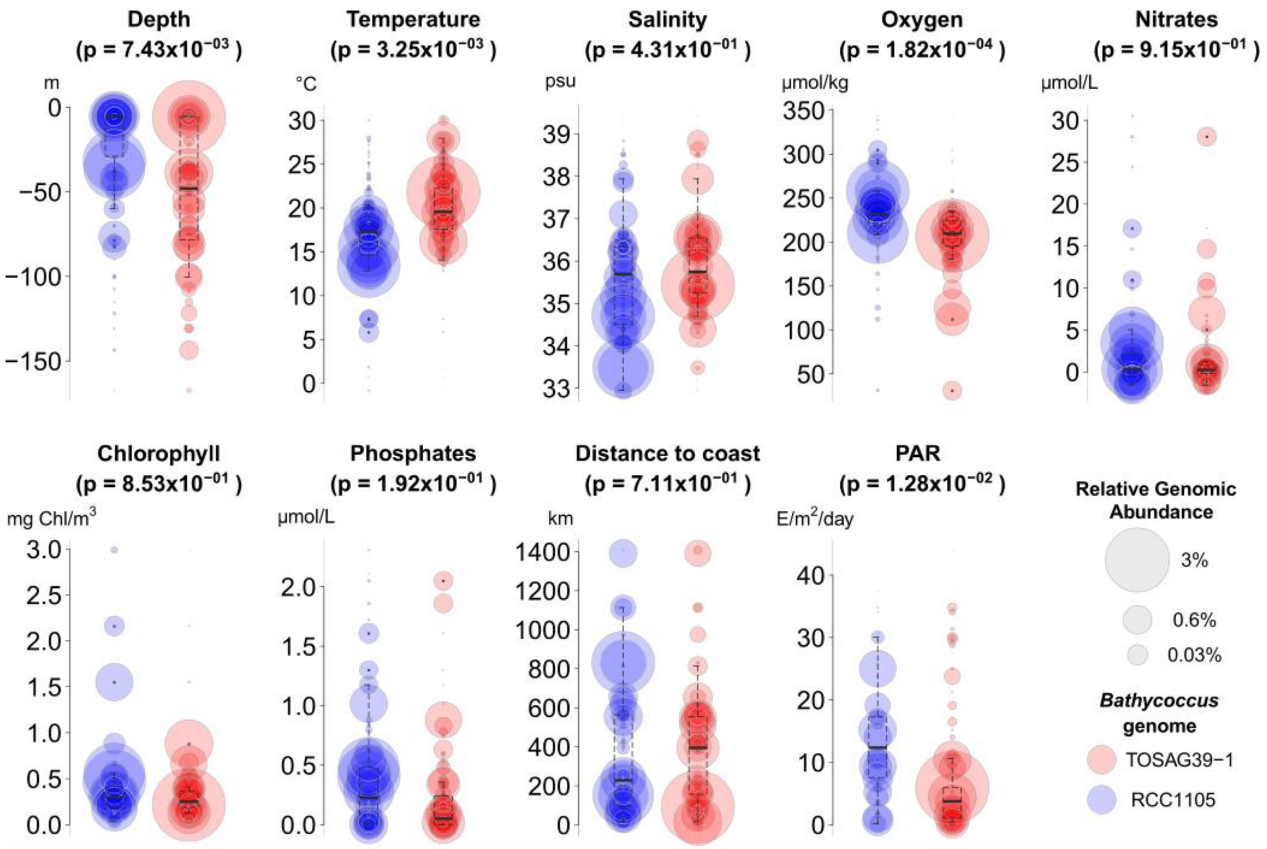

Figure S10. Environmental parameters and genomic abundances of *Bathycoccus*. PAR (Photosynthetically Active Radiation) corresponds to AMODIS satellite data for surface samples and to computed estimations for DCM samples. Temperature, oxygen, depth and light are parameters that gave significantly different distributions between the two *Bathycoccus* (Wilcoxon probability values). Sizes of circles are proportional to relative metagenomics abundance, according to the scale given in the legend. Boxplots over bubble plots indicate organism range distribution within samples containing high abundances of *Bathycoccus*, without taking in account abundance values.

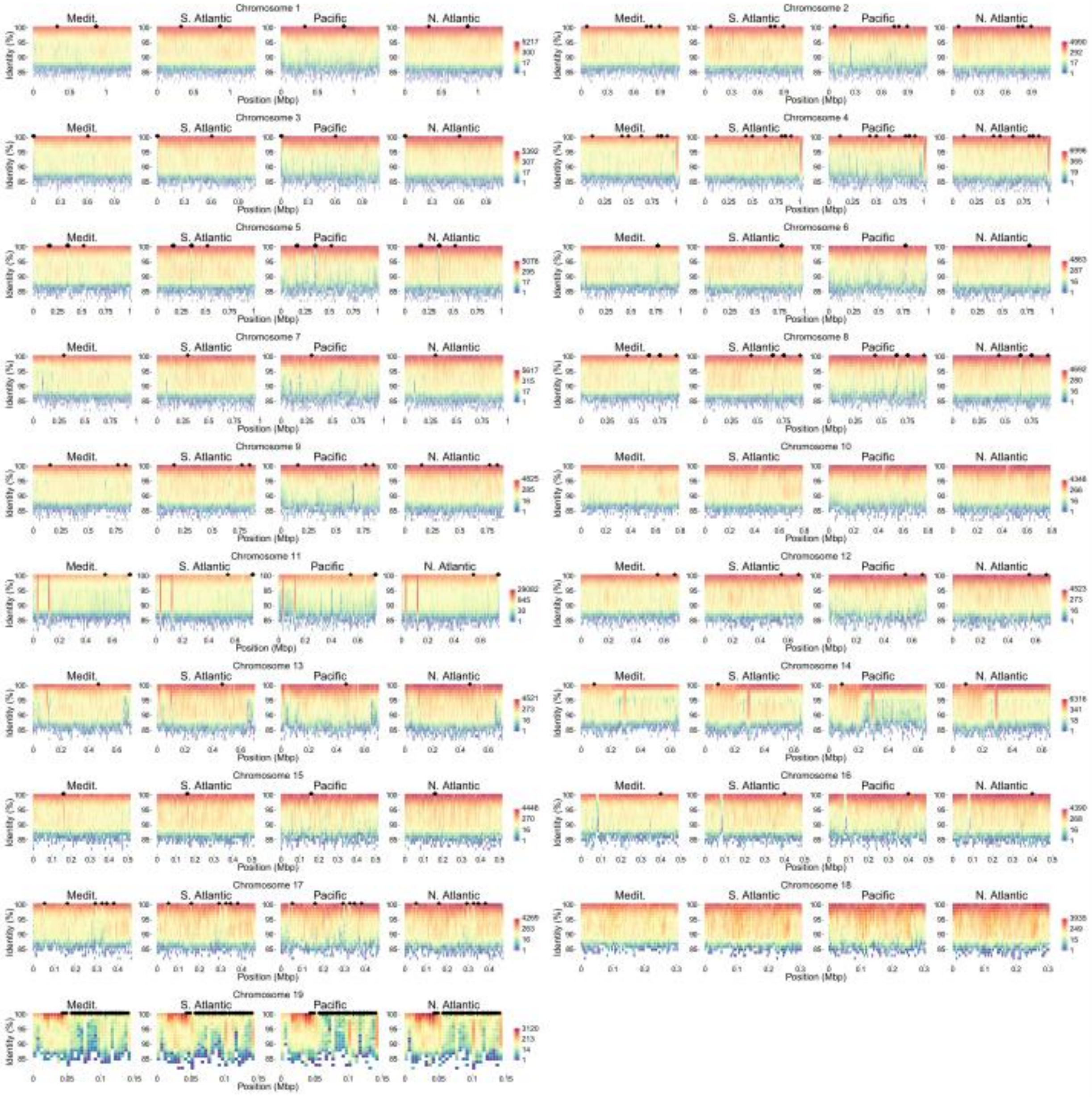

Figure S11. Metagenomic fragment recruitment plot on all chromosomes separated by large marine basins. Chromosome positions of dispensable genes are indicated by black dots. Gradient colors correspond to density of recruited metagenomic reads from low (blue) to high (red).

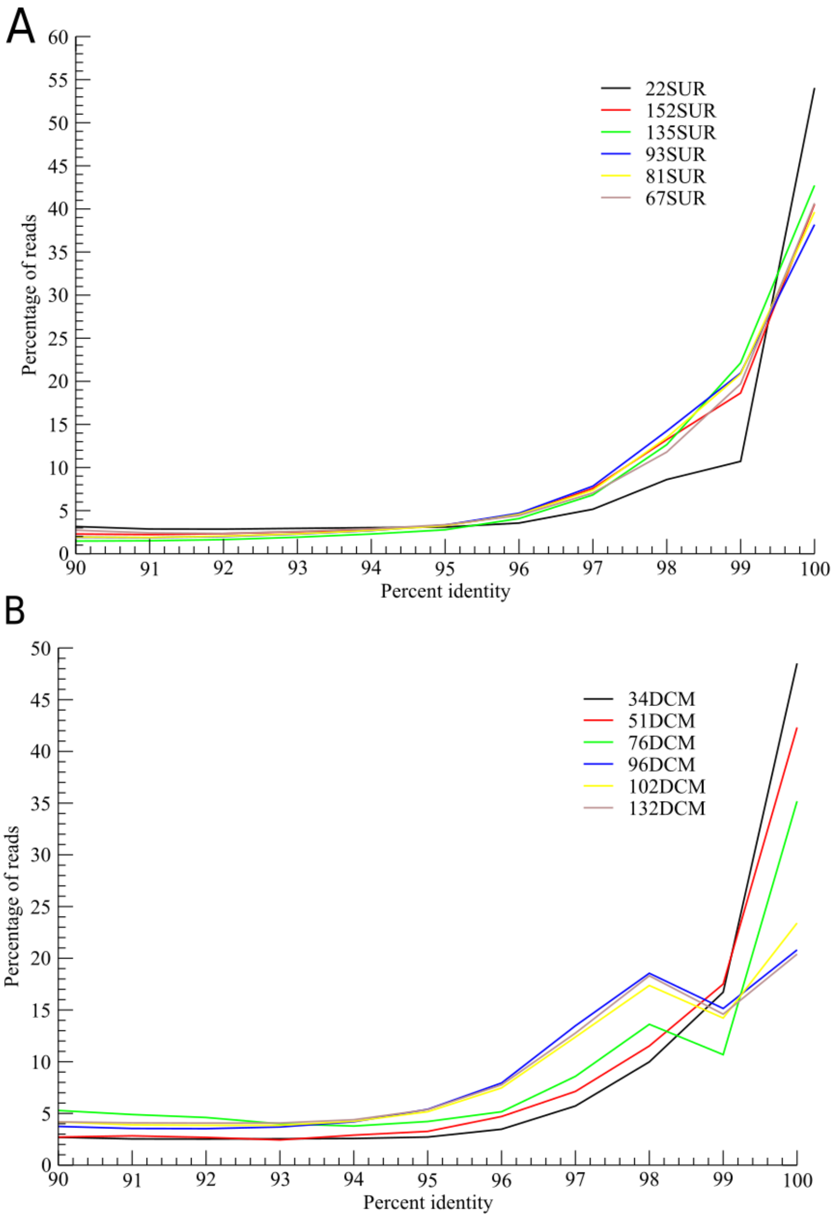

Figure S12. Distribution of identity percent of *Tara* Oceans metagenomic reads mapped onto RCC1105 genome (A) and TOSAG39-1 assembly (B). We only used *Tara* Oeans samples where the presence of only one *Bathycoccus* genome was detected.

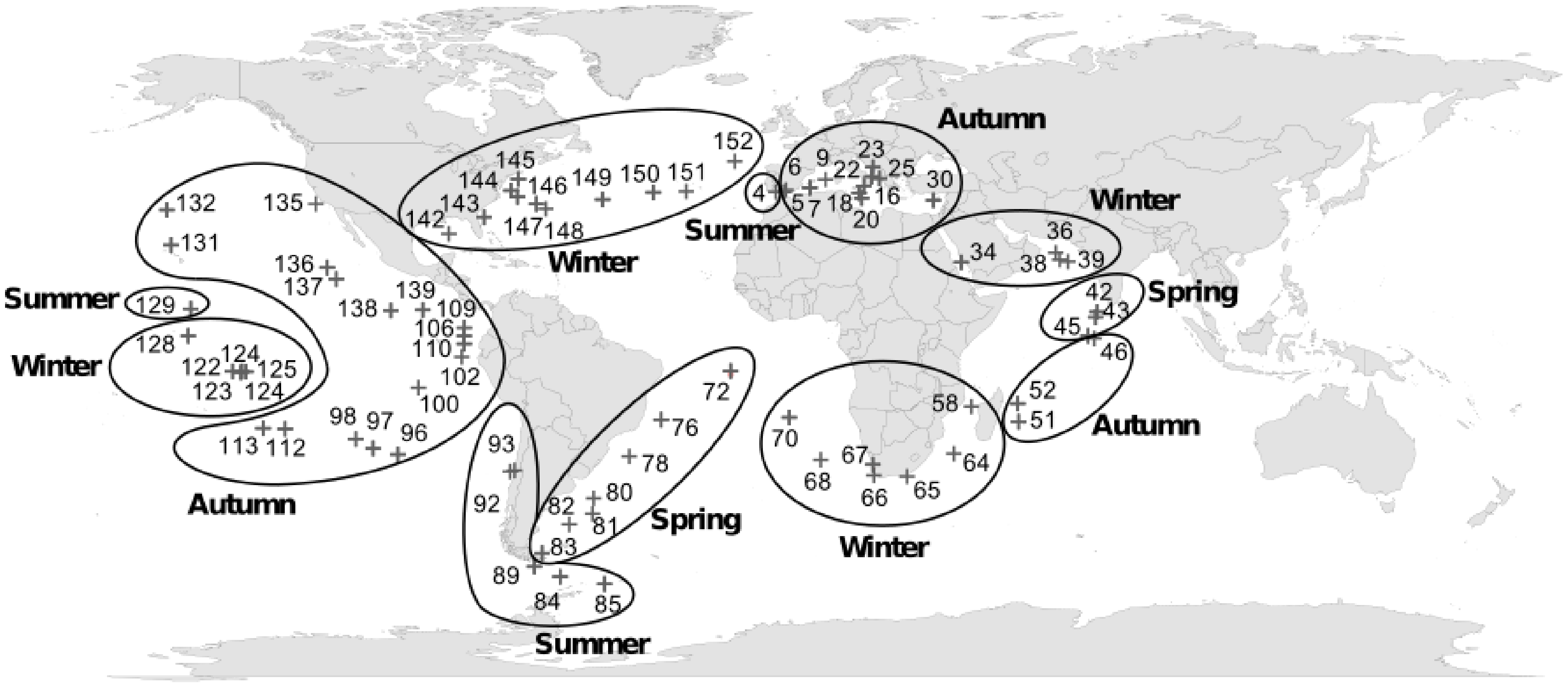

Figure S13. Map of the stations of the *Tara* Oceans expedition with seasons when sampled. This map was created using R-package maps_2.1-6, mapproj_1.1-8.3, gplots_2.8.0 and mapplots_1.4 (version R-2.13, https://cran.r-project.org/web/packages/maps/index.html).

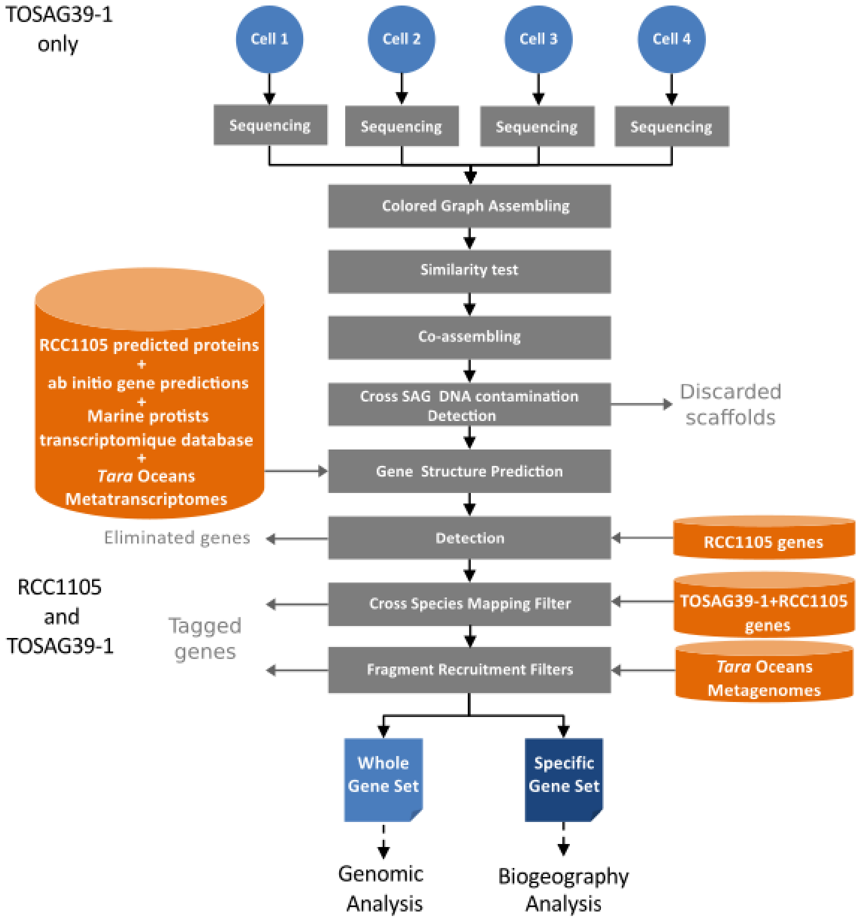

Figure S14. Pipeline for data acquisition and quality control.

Table S1. Comparisons of gene features of the two *Bathycoccus* gene sets.

**Characterization**		**RCC1105 Genes**			**RCC1105 Genes (except chromosome 19)**			**TOSAG39-1 Predicted Genes**		
		**All**	**Dispensable**	**Not Dispensable**	**All**	**Dispensable**	**Not Dispensable**	**All**	**Dispensable**	**Not Dispensable**
Gene number		7807	108	7699	7735	58	7677	6157	106	6051
Gene size (nt) mean. : sd		1609.36 : 1281	1014.19 : 942	1617.70 : 1287	1613.30 : 1287	1137.43 : 1049	1616.90 : 1287	1344.62 : 1074	511.89 : 392	1359.21 : 1087
Monoexonic Genes		6648 (85%)	100 (93%)	6548 (85%)	6585	52 (90%)	6533 (85%)	4596 (75%)	75 (71%)	4521 (75%)
Number of exons mean : sd		1.19 : 1	1.08 : 1	1.19 : 1	1.19 : 1	1.10 : 1	1.19 : 1	1.33 : 1	1.30 : 1	1.33 : 1
CDS length (nt) mean : sd		1578.44 : 1251	1006.78 : 939	1586.45 : 1257	1582.22 : 1257	1126.76 : 1026	1585.66 : 1257	1242.16 : 984	455.12 : 330	1255.95 : 999
Number of introns		1504	9	1495	1494	6	1488	2028	32	1996
Introns Size (nt) mean : sd		160.50 : 131	88.89 : 57	160.93 : 132	160.92 : 131	103.17 : 44	161.15 : 132	217.25 : 154	101.88 : 83	219.10 : 154
Metagenomic Abundance (*a*) (RPKM values)	All Samples. mean. : sd	2.47 : 1.16	0.44 : 0.69	2.50 : 1.14	2.49 : 1.14	0.56 : 0.82	2.51 : 1.13	3.28 : 1.34	0.50 : 0.73	3.33 : 1.30
	Samples with detected signal only. mean. : sd	2.49 : 1.14	0.75 : 0.76	2.51 : 1.13	2.50 : 1.13	0.92 : 0.88	2.51 : 1.13	3.31 : 1.31	0.82 : 0.78	3.34 : 1.29
Metatranscriptomic Abundance (*b*) (RPKM values)	All Samples. Mean. : sd	1.34 : 1.40	0.15 : 0.46	1.36 : 1.41	1.35 : 1.41	0.16 : 0.55	1.68 : 1.64	1.64 : 1.64	0.12 : 0.36	1.71 : 1.64
	Samples with detected signal only. Mean. : sd	1.58 : 1.39	0.58 : 0.76	1.58 : 1.39	1.58 : 1.40	0.70 : 0.96	2.04 : 1.59	2.03 : 1.59	0.67 : 0.59	2.05 : 1.59
Relative Transcriptomic Activity (a / b)	All Samples. mean. : sd	0.47 : 0.71	0.20 : 0.55	0.47 : 0.71	0.47 : 0.71	0.18 : 0.55	0.47 : 0.71	0.49 : 0.73	0.13 : 0.43	0.49 : 0.73
	Samples with detected signal only. mean. : sd	0.56 : 0.74	0.77 : 0.84	0.56 : 0.74	0.56 : 0.74	0.73 : 0.89	0.56 : 0.74	0.59 : 0.76	0.72 : 0.78	0.59 : 0.76

RPKM: reads per kilobase of transcript per million reads mapped.

Table S2. Depths of the Mixed Layer Depth (MLD) and of samples from the Deep Chlorophyll Maximum (DCM; italic red correspond to DCM samples taken above the MLD) for each *Tara* Ocean station used in this paper.

***Tara* Oceans Station**	**DCM sample depths (m)**	**MLD (m)**
**4**	39	4
**7**	42	18
**8**	45	3
**9**	55	21
**18**	62	39
**22**	31	9
**23**	55	9
**25**	52	29
**30**	69	41
**34**	60	26
**36**	17	7
**38**	25	11
**39**	25	9
**42**	79	21
**51**	80	40
**52**	79	47
**58**	67	17
64	*64*	*71*
65	*29*	*47*
66	*29*	*90*
68	*40*	*187*
**72**	95	75
**76**	148	34
**78**	118	34
**80**	83	12
**81**	38	29
**82**	42	29
**85**	87	38
**93**	34	22
**96**	153	42
**97**	174	50
**98**	183	53
**100**	58	35
**102**	46	18

**Tara Oceans Station**	**DCM sample depths (m)**	**MLD (m)**
**106**	47	12
**109**	30	9
**110**	49	22
**112**	154	131
**122**	113	71
**125**	138	95
**128**	42	35
**129**	85	76
**131**	109	36
**132**	114	41
**135**	30	13
**137**	44	17
**138**	58	24
142	*124*	*142*
143	*49*	*69*
150	*40*	*77*
**151**	78	36

Table S3. Annotations of the RCC1105 dispensable genes that have functional predictions.

Pfam	Note	Gene Identifier	Number of Dispensable Genes	
			Whole Genome	Chromosome 19
Pfam14312	FG-GAP repeat	Bathy02g04860	1	0
Pfam13465	Zinc-finger double domain	Bathy04g03240, Bathy04g03240, Bathy09g04110, Bathy09g04110	4	0
Pfam00808	Histone-like transcription factor (CBF/NF-Y) and archaeal histone	Bathy04g04090	1	0
Pfam06977	SdiA-regulated	Bathy04g04270	1	0
Pfam07727	Reverse transcriptase (RNA-dependent DNA polymerase)	Bathy04g04610, Bathy19g00670	2	1
Pfam01844	HNH endonuclease	Bathy05g02900	1	0
pfam12796	Ankyrin repeats (3 copies)	Bathy07g01420, Bathy12g03030	2	0
pfam14099	Polysaccharide lyase	Bathy08g04110	1	0
pfam01866	Putative diphthamide synthesis protein	Bathy08g04120	1	0
pfam03382	Mycoplasma protein of unknown function, DUF285	Bathy17g01470, Bathy17g01550	2	0
pfam11913	Protein of unknown function (DUF3431)	Bathy19g00310	1	1

pfam13383	Methyltransferase domain	Bathy19g00340, Bathy19g00540	2	2
pfam13578	Methyltransferase domain	Bathy19g00410	1	1
pfam00777	Glycosyltransferase family 29 (sialyltransferase)	Bathy19g00420	1	1
pfam04321	RmlD substrate binding domain	Bathy19g00510	1	1
pfam13489	Methyltransferase domain	Bathy19g00590	1	1

Table S4. Dispensable genes of RCC1105.

Bathy01g01790	Bathy08g04120	Bathy19g00350
Bathy01g04690	Bathy08g04130	Bathy19g00360
Bathy01g04700	Bathy08g04940	Bathy19g00370
Bathy02g00365	Bathy09g00830	Bathy19g00380
Bathy02g04020	Bathy09g04110	Bathy19g00390
Bathy02g04230	Bathy09g04450	Bathy19g00400
Bathy02g04860	Bathy11g02890	Bathy19g00410
Bathy03g00010	Bathy11g03900	Bathy19g00420
Bathy03g00030	Bathy11g03920	Bathy19g00430
Bathy03g00040	Bathy12g03030	Bathy19g00440
Bathy03g03150	Bathy12g03670	Bathy19g00450
Bathy04g00740	Bathy13g02130	Bathy19g00460
Bathy04g02210	Bathy14g00440	Bathy19g00470
Bathy04g02620	Bathy15g00910	Bathy19g00480
Bathy04g03240	Bathy16g02050	Bathy19g00490
Bathy04g04090	Bathy17g00250	Bathy19g00510
Bathy04g04270	Bathy17g00780	Bathy19g00520
Bathy04g04280	Bathy17g01470	Bathy19g00530
Bathy04g04610	Bathy17g01550	Bathy19g00540
Bathy05g00940	Bathy17g01690	Bathy19g00550
Bathy05g00970	Bathy17g01840	Bathy19g00560
Bathy05g00980	Bathy19g00160	Bathy19g00570
Bathy05g02010	Bathy19g00175	Bathy19g00580
Bathy05g02020	Bathy19g00200	Bathy19g00590

Bathy05g02030	Bathy19g00230	Bathy19g00600
Bathy05g02040	Bathy19g00240	Bathy19g00610
Bathy05g02900	Bathy19g00250	Bathy19g00620
Bathy06g04070	Bathy19g00260	Bathy19g00630
Bathy06g04080	Bathy19g00270	Bathy19g00640
Bathy06g04090	Bathy19g00280	Bathy19g00650
Bathy07g01420	Bathy19g00290	Bathy19g00660
Bathy08g02440	Bathy19g00300	Bathy19g00670
Bathy08g03500	Bathy19g00310	Bathy19g00680
Bathy08g03510	Bathy19g00320	Bathy19g00690
Bathy08g03520	Bathy19g00330	Bathy19g00700
Bathy08g04110	Bathy19g00340	

Table S5. Dispensable genes of TOSAG39-1.

TOSAG39-1_gene78	TOSAG39-1_gene2608	TOSAG39-1_gene4518
TOSAG39-1_gene145	TOSAG39-1_gene2703	TOSAG39-1_gene4704
TOSAG39-1_gene223	TOSAG39-1_gene2704	TOSAG39-1_gene4784
TOSAG39-1_gene226	TOSAG39-1_gene2878	TOSAG39-1_gene4883
TOSAG39-1_gene229	TOSAG39-1_gene2935	TOSAG39-1_gene5106
TOSAG39-1_gene278	TOSAG39-1_gene2982	TOSAG39-1_gene5107
TOSAG39-1_gene358	TOSAG39-1_gene2987	TOSAG39-1_gene5131
TOSAG39-1_gene382	TOSAG39-1_gene3033	TOSAG39-1_gene5174
TOSAG39-1_gene383	TOSAG39-1_gene3035	TOSAG39-1_gene5178
TOSAG39-1_gene394	TOSAG39-1_gene3051	TOSAG39-1_gene5189
TOSAG39-1_gene509	TOSAG39-1_gene3339	TOSAG39-1_gene5291
TOSAG39-1_gene521	TOSAG39-1_gene3340	TOSAG39-1_gene5327
TOSAG39-1_gene588	TOSAG39-1_gene3341	TOSAG39-1_gene5480
TOSAG39-1_gene615	TOSAG39-1_gene3361	TOSAG39-1_gene5523
TOSAG39-1_gene616	TOSAG39-1_gene3460	TOSAG39-1_gene5695
TOSAG39-1_gene791	TOSAG39-1_gene3505	TOSAG39-1_gene5721
TOSAG39-1_gene993	TOSAG39-1_gene3508	TOSAG39-1_gene5791
TOSAG39-1_gene997	TOSAG39-1_gene3562	TOSAG39-1_gene5792
TOSAG39-1_gene1003	TOSAG39-1_gene3690	TOSAG39-1_gene5901
TOSAG39-1_gene1004	TOSAG39-1_gene3830	TOSAG39-1_gene5902
TOSAG39-1_gene1048	TOSAG39-1_gene3846	TOSAG39-1_gene5986
TOSAG39-1_gene1113	TOSAG39-1_gene3880	TOSAG39-1_gene5987
TOSAG39-1_gene1178	TOSAG39-1_gene3915	TOSAG39-1_gene6023
TOSAG39-1_gene1388	TOSAG39-1_gene3958	TOSAG39-1_gene6026

TOSAG39-1_gene1392	TOSAG39-1_gene3959	TOSAG39-1_gene6027
TOSAG39-1_gene1403	TOSAG39-1_gene3966	TOSAG39-1_gene6079
TOSAG39-1_gene1416	TOSAG39-1_gene3967	TOSAG39-1_gene6104
TOSAG39-1_gene1417	TOSAG39-1_gene3972	TOSAG39-1_gene6187
TOSAG39-1_gene1483	TOSAG39-1_gene4016	TOSAG39-1_gene6188
TOSAG39-1_gene1694	TOSAG39-1_gene4042	TOSAG39-1_gene6222
TOSAG39-1_gene1740	TOSAG39-1_gene4043	TOSAG39-1_gene6362
TOSAG39-1_gene1751	TOSAG39-1_gene4060	TOSAG39-1_gene6376
TOSAG39-1_gene1765	TOSAG39-1_gene4062	TOSAG39-1_gene6422
TOSAG39-1_gene1818	TOSAG39-1_gene4273	TOSAG39-1_gene6426
TOSAG39-1_gene2202	TOSAG39-1_gene4303	TOSAG39-1_gene6440
TOSAG39-1_gene2203	TOSAG39-1_gene4517	

Table S6. Summary of the matches obtained with the discarded scaffolds of TOSAG39-1 assembly.

**Match**	**Proportion**
No match	42.4%
Bathycoccus prasinos	37.8%
Bacteria	10.8%
Mitochondrion	3.6%
Cyprinus carpio	0.6%
Chloroplast	0.5%
BpV2 virus	0.4%
Bacteriophage S13	0.2%
Other	3.8%

References

1. Moreau, H. *et al.* Gene functionalities and genome structure in *Bathycoccus prasinos* reflect cellular specializations at the base of the green lineage. *Genome Biol.* **13,** R74 (2012).
2. Sterck, L., Billiau, K., Abeel, T., Rouzé, P. & Van de Peer, Y. ORCAE: online resource for community annotation of eukaryotes. *Nat. Methods* **9,** 1041–1041 (2012).
3. Vaulot, D. *et al.* Metagenomes of the picoalga *Bathycoccus* from the Chile coastal upwelling. *PLoS ONE* **7,** e39648 (2012).
4. Monier, A. *et al.* Phosphate transporters in marine phytoplankton and their viruses: cross-domain commonalities in viral-host gene exchanges. *Environ. Microbiol.* **14,** 162–176 (2012).
5. Stepanauskas, R. & Sieracki, M. E. Matching phylogeny and metabolism in the uncultured marine bacteria, one cell at a time. *Proc. Natl. Acad. Sci. U. S. A.* **104,** 9052–9057 (2007).
6. Edgar, R. C. MUSCLE: multiple sequence alignment with high accuracy and high throughput. *Nucleic Acids Res.* **32,** 1792–1797 (2004).
7. Movahedi, N. S., Forouzmand, E. & Chitsaz, H. De novo co-assembly of bacterial genomes from multiple single cells. in *2012 IEEE International Conference on Bioinformatics and Biomedicine (BIBM)* 1–5 (2012). doi:10.1109/BIBM.2012.6392618
8. Bankevich, A. *et al.* SPAdes: a new genome assembly algorithm and its applications to single-cell sequencing. *J. Comput. Biol. J. Comput. Mol. Cell Biol.* **19,** 455–477 (2012).
9. Boetzer, M., Henkel, C. V., Jansen, H. J., Butler, D. & Pirovano, W. Scaffolding pre-assembled contigs using SSPACE. *Bioinforma. Oxf. Engl.* **27,** 578–579 (2011).
10. Luo, R. *et al.* SOAPdenovo2: an empirically improved memory-efficient short-read de novo assembler. *GigaScience* **1,** 18 (2012).

11. Li, W. & Godzik, A. Cd-hit: a fast program for clustering and comparing large sets of protein or nucleotide sequences. *Bioinforma. Oxf. Engl.* **22,** 1658–1659 (2006).

12. Fu, L., Niu, B., Zhu, Z., Wu, S. & Li, W. CD-HIT: accelerated for clustering the next-generation sequencing data. *Bioinformatics* **28,** 3150–3152 (2012).

13. Smit, A., Hubley, R. & Green, P. RepeatMasker Open-4.0 at http://www.repeatmasker.org. (2013).

14. Altschul, S. F., Gish, W., Miller, W., Myers, E. W. & Lipman, D. J. Basic local alignment search tool. *J. Mol. Biol.* **215,** 403–410 (1990).

15. Suzek, B. E., Huang, H., McGarvey, P., Mazumder, R. & Wu, C. H. UniRef: comprehensive and non-redundant UniProt reference clusters. *Bioinforma. Oxf. Engl.* **23,** 1282–1288 (2007).

16. Keeling, P. J. *et al.* The Marine Microbial Eukaryote Transcriptome Sequencing Project (MMETSP): illuminating the functional diversity of eukaryotic life in the oceans through transcriptome sequencing. *PLOS Biol* **12,** e1001889 (2014).

17. Korf, I. Gene finding in novel genomes. *BMC Bioinformatics* **5,** 59 (2004).

18. Denoeud, F. *et al.* Annotating genomes with massive-scale RNA sequencing. *Genome Biol.* **9,** R175 (2008).

19. Smith, T. F. & Waterman, M. S. Identification of common molecular subsequences. *J. Mol. Biol.* **147,** 195–197 (1981).

20. Kurtz, S. *et al.* Versatile and open software for comparing large genomes. *Genome Biol.* **5,** R12 (2004).

21. Rutherford, K. *et al.* Artemis: sequence visualization and annotation. *Bioinforma. Oxf. Engl.* **16,** 944–945 (2000).

22. Monier, A., Sudek, S., Fast, N. M. & Worden, A. Z. Gene invasion in distant eukaryotic lineages: discovery of mutually exclusive genetic elements reveals marine biodiversity. *ISME J.* **7,** 1764–1774 (2013).

23. Langmead, B. & Salzberg, S. L. Fast gapped-read alignment with Bowtie 2. *Nat. Methods* **9,** 357–359 (2012).

24. Wickham, H. *ggplot2*. (Springer New York, 2009).

25. Pruitt, K. D., Tatusova, T. & Maglott, D. R. NCBI Reference Sequence (RefSeq): a curated non-redundant sequence database of genomes, transcripts and proteins. *Nucleic Acids Res.* **33,** D501–D504 (2005).

26. Montero Manso, P. & Vilar, A. TSclust: An R Package for Time Series Clustering. *Journal of Statistical Software* 1–43 (2014).

27. Morgulis, A., Gertz, E. M., Schäffer, A. A. & Agarwala, R. A fast and symmetric DUST implementation to mask low-complexity DNA sequences. *J. Comput. Biol. J. Comput. Mol. Cell Biol.* **13,** 1028–1040 (2006).

28. de Vargas, C. *et al.* Eukaryotic plankton diversity in the sunlit ocean. *Science* **348,** 1261605 (2015).

29. Pesant, S. *et al.* Open science resources for the discovery and analysis of Tara Oceans data. *Sci. Data* **2,** 150023 (2015).

30. Morel, A. *et al.* Examining the consistency of products derived from various ocean color sensors in open ocean (Case 1) waters in the perspective of a multi-sensor approach. *Remote Sens. Environ.* **111,** 69–88 (2007).

31. Marchler-Bauer, A. *et al.* CDD: NCBI's conserved domain database. *Nucleic Acids Res.* **43,** D222–226 (2015).
